# Sec10 negatively regulates antiviral immunity by downregulating NRF2-ATF4-RIG-I axis

**DOI:** 10.7150/ijbs.117430

**Published:** 2025-09-03

**Authors:** Peili Hou, Fuzhen Zhang, Xiaonan Sun, Hongchao Zhu, Yueyue Feng, Jun Wang, Xiaoyun Wang, Yuanyuan Han, Rui Li, Chuanhong Wang, Yingying Li, Hongmei Wang, Hongbin He

**Affiliations:** Ruminant Diseases Research Center, College of Life Sciences, Shandong Normal University, Jinan, 250358, People's Republic of China.

**Keywords:** Sec10, RIG-I, transcriptional regulation, antiviral immunity

## Abstract

Sec10, as a central component of the eight-protein exocyst complex, plays a crucial role in the exocytosis. However, its role in antiviral immunity has remained elusive. Here, we discover that Sec10 negatively regulates antiviral immune response by downregulation of RIG-I at transcriptional level, thereby facilitating RNA replication in multiple cells. Mechanistically, we demonstrate that ATF4 binds to the RIG-I promoter and promotes RIG-I transcription, and NRF2 upregulates ATF4 activity and expression. Notably, Sec10 triggers the inactivation of the NRF2-ATF4 axis during RNA viral infection, which is, in turn restrains RIG-I transcription, attenuating antiviral IFN-I response. Importantly, Sec10 deficiency results in enhanced innate immunity, diminished SeV load and morbidity in mice. Taken together, we firstly unveil the function of Sec10 in viral infection, and elucidate its novel mechanisms of antiviral immunity via the NRF2-ATF4-RIG-I axis, which provides the potential therapeutic targets and offers new insights for antiviral drug development.

## Introduction

Emerging and re-emerging RNA viruses with high variability and adaptability allows them to spread between humans and animals, occasionally causing epidemics and pandemics worldwide, which seriously threaten human and animal health and social development^1^, ^2^. Despite extensive efforts by the research community to identify therapeutic antiviral agents to address such emergencies, the specificity to individual viruses and the potential mutagenicity have constrained the development of clinically targeted and effective drugs^3^. Thus, elucidating virus-host interactions and identifying novel biological targets that exhibit broad-spectrum antiviral activity are critical for advancing the development of innovative and highly effective antiviral therapeutics.

Antiviral innate immune response represents a critical line of defense against viral infections. Upon viral infection, host pattern recognition receptors (PRRs), including RIG-I-like receptors (RLRs), cyclic GMP-AMP synthase (cGAS)-stimulator of interferon genes (STING), Toll-like receptors (TLRs), and NOD-like receptors (NLRs) recognize primary pathogen-associated molecular patterns (PAMPs) such as viral nucleic acids (e.g., 5′-phosphorylated RNA, viral DNAs or double-stranded RNA), thereby initiating a cascade of signal transduction events to regulate innate immune responses against pathogens^4^, ^5^.

Specifically, in the battle between RNA viruses and their hosts, it was widely known that retinoic acid inducible gene I (RIG-I) functions as a cytosolic pattern recognition receptor that initiates innate antiviral immunity by detecting exogenous viral RNAs^6^, ^7^. Upon sensing viral RNA (e.g., 5' triphosphate double stranded RNA (5' ppp-dsRNA)), RIG-I undergoes conformational changes that expose its caspase activation and recruitment domains to interact with the downstream adaptor mitochondrial antiviral signaling protein (MAVS). This interaction in turn leads to the subsequent activation of interferon regulatory factors (IRF3, IRF7 and NF-κB), ultimately driving the production of type I interferon (IFN-I) and pro-inflammatory cytokines to orchestrate antiviral immunity^8^, ^9^. A growing literature demonstrates that RIG-I activation has to be tightly regulated to ensure effective virus inhibition while minimizing an excessive inflammatory response^10^. However, invading viruses have developed multiple strategies to overcome host antiviral immune signaling, of which transcriptional regulation is one of the most important^11^. Interferon gamma inducible protein 16 (IFI16) positively upregulates RIG-I transcription through direct binding to and recruitment of RNA polymerase II to the RIG-I promoter^12^. Interferon regulatory factor-1 (IRF-1) positively regulates interferon- or dsRNA-induced RIG-I transcription, and plays an essential role in anti-viral immunity^13^. CCAAT enhancer binding protein beta (C/EBPβ) is an important transcription factor for RIG-I transcriptional regulation via forming complex with viral NS1 protein during influenza virus infection^14^. Ubiquitin protein ligase E3 component N-recognin 5 (UBR5) enables rapid upregulation of RLR expression including RIG-I and MDA5 to boost antiviral immune responses by ubiquitinating and de-SUMOylating tripartite motif protein 28 (TRIM28)^15^. However, the underlying mechanisms of RIG-I transcriptional regulation during viral infection remain largely unknown.

The exocyst complex is an evolutionarily conserved 750-kDa heterooctameric protein complex composed of Sec3, Sec5, Sec6, Sec8, Sec10, Sec15, Exo70 and Exo84^16^. It is well known for targeting and docking secretory vesicles carrying proteins to the plasma membrane^17^. A central component of the exocyst is Sec10 (also known as Exoc5), which is thought to serve as a “linker” or central hub for bridging the other components of exocyst complex^18^. Sec10 has a broad tissue distribution at both the protein and mRNA levels. Although Sec10 has a wide range of reported functions including tubulogenesis, neurite growth, endosome recycling, translocon, cell migration, ciliogenesis, cystogenesis, epidermal growth factor receptor (EGFR), endocytosis, which may be dependent or independent on its fundamental role in exocytosis^19^-^21^. However, its role in viral infection and antiviral immunity has remained elusive. We further investigated the protein levels of Sec10 were increased in the early phase of Sendai virus (SeV) infection at 2, 4, 6 hpi (Fig.S1), suggesting that Sec10 might be involved in viral replication and antiviral immune response.

In this study, we identified Sec10 as a negative modulator of RIG-I-dependent antiviral signaling against RNA infection including SeV, Influenza A virus subtype H1N1 and bovine ephemeral fever virus (BEFV) *in vitro* and *in vivo*. Further study indicated that Sec10 suppressed activating transcription factor 4 (ATF4)-mediated RIG-I transcriptional expression through down-regulation of nuclear factor erythroid 2-related factor 2 (NRF2). Overall, our findings unveil a novel role of Sec10 in the antiviral immune response. Specifically, Sec10 downregulates the NRF2-ATF4-RIG-I signaling axis, leading to a decrease in the IFN-I response against RNA virus infection. This discovery provides valuable insight into the development of targeted antiviral agents.

## Results

### Sec10 promotes SeV, H1N1 and BEFV replication

**Figure 1:**
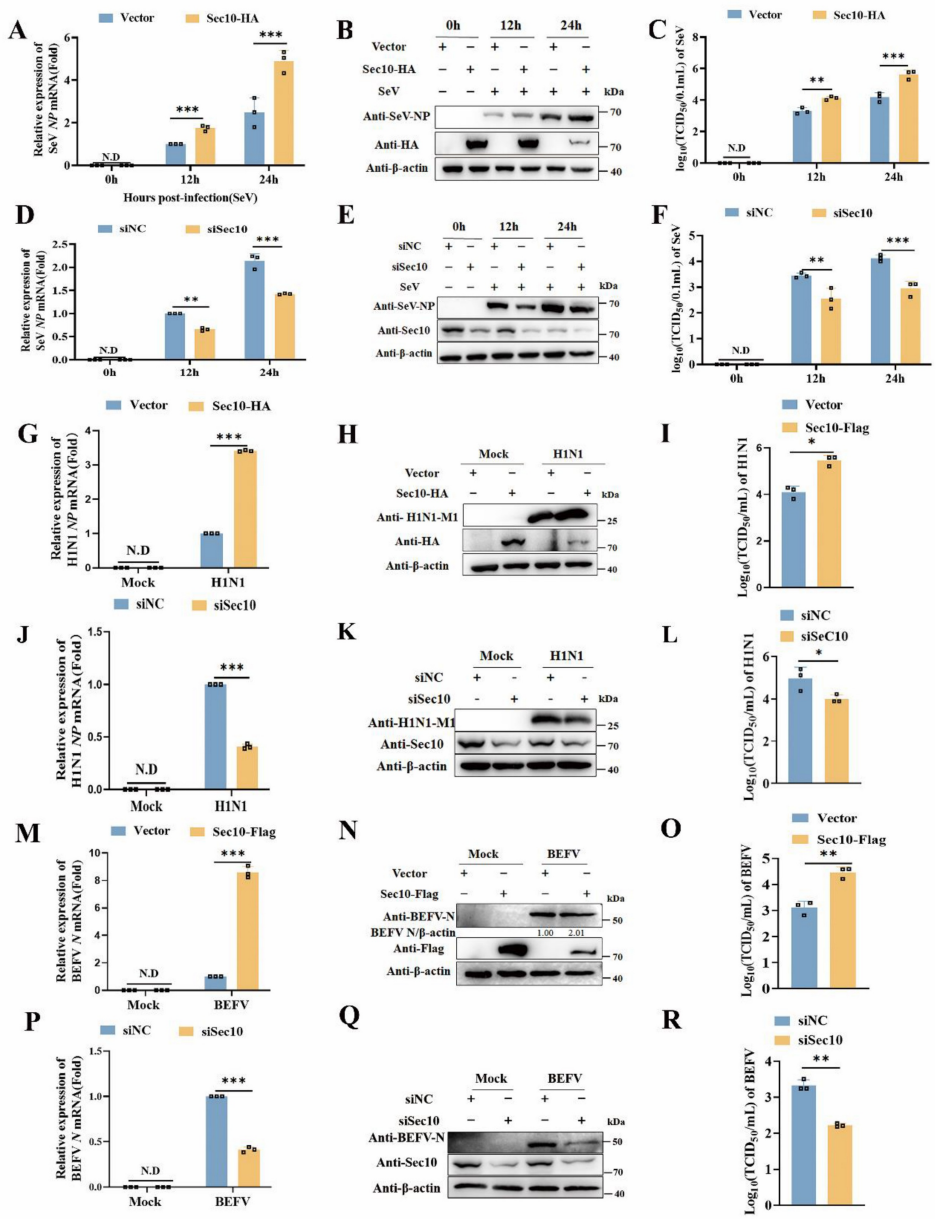
** Sec10 promotes SeV, H1N1 and BEFV replication.** (**A-F**) Real-time PCR and Western blotting analysis of the expression levels of the SeV *NP* genes (A, D) and NP proteins (B, E) and TCID_50_ assays of the viral titer (C, F) in SeV(100IAU/mL)-infected Sec10-overexpressing (Sec10-HA) or control (vector) HeLa cells, or in HeLa cells transfected with Sec10-specific siRNAs (siSec10) or scrambled siRNA (siNC), and infected with SeV (100IAU/mL) for the indicated durations. (**G-L**) Real-time PCR and Western blotting analysis of the expression levels of the *NP* gene (G, J) and M1 protein (H, K) of H1N1 and TCID_50_ analysis of the viral titer (I, L) in the H1N1-infected Sec10- overexpressing (Sec10-HA) or control (Vector) A549 cell lines, Sec10-silenced(siSec10) or control (siNC) A549 cells for 12 h. (**M-R**) Real-time PCR and Western blotting analysis of the expression levels of the BEFV *N* genes (M, P) and N proteins (N, Q) and TCID_50_ analysis of the viral titer (O, R) in the Sec10- overexpressing (Sec10-Flag) or control (Vector) MDBK cell lines, Sec10-silenced (siSec10) or control (siNC) MDBK cells, and infected with BEFV (MOI=0.1) for 12 h. Data in (B, E, H, K, N and Q) are representative one of three independent experiments. Data in (A, C, D, F, G, I, J, L, M, O, P and R) are presented as mean±S.D from three independent experiments, two-way ANOVA for date in (A, C, D and F), and two-tailed Student's t test for date in (G, I, J, L, M, O, P and R). N.D, not detected. *P<0.05, **P<0.01, and ***P<0.001.

To evaluate the effect of Sec10 in viral infection, viral replication was determined in multiple passaged cells via overexpressing and silencing of Sec10. As shown in Fig. 1A-C, ectopic expression of Sec10 in HeLa cells resulted in a concomitant increase in SeV replication, as demonstrated by increased SeV *NP* mRNA and NP protein expression, and virus titer of SeV. Consistent with the results, the knockdown of Sec10 in HeLa cells reduced SeV replication (Fig. 1D-F). Furthermore, overexpression of Sec10 in H1N1-infected A549 cells increased *NP* gene, M1 protein and viral titer of H1N1(Fig. 1G-I), whereas the knockdown of Sec10 in H1N1-infected A549 cells had the opposite effect (Fig. 1J-L). Accordingly, Sec10 overexpression sharply promoted BEFV propagation in MDBK cells, as reflected by enhanced viral *N* gene, N protein expression, and virus titer after infection with BEFV (Fig. 1 M-O). In contrast, knocking down Sec10 dramatically curtailed BEFV propagation (Fig. 1P-R). Taken together, these results indicate that Sec10 positively regulates the replication of multiple RNA viruses in various passaged cells.

### Sec10 suppresses innate immune responses during RNA viral infection

**Figure 2:**
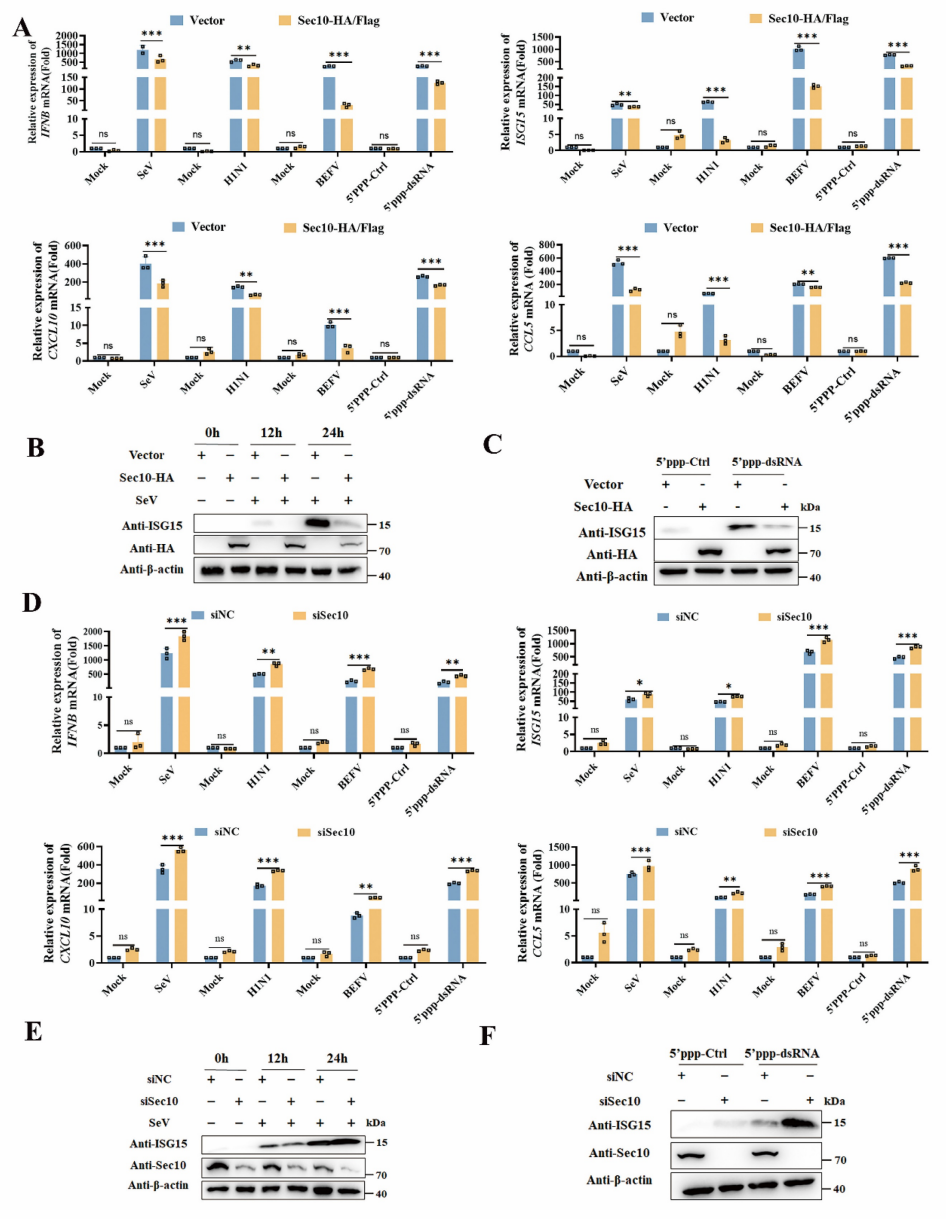
** Sec10 suppresses RNA virus-induced type I IFN signaling pathway in passaged cells.** (**A**) Real-time PCR analysis of* IFNB*, *ISG15*, *CXCL10* and *CCL5* mRNA expression in Sec10-overexpressing HeLa cell lines (Sec10-HA) infected with SeV, A549 cell lines(Sec10-HA) infected with H1N1 and MDBK cell lines(Sec10-Flag) with BEFV infection for 12 h, or Sec10-overexpressing HeLa cell lines transfected with 5'ppp-dsRNA for 8 h. 5'ppp-dsRNA control (5'ppp-Ctrl) is a 19 mer blunt-end dsRNA without a 5'triphosphate (InvivoGen, tlrl-3prnac). (**B, C**) Immunoblot analysis of the protein expression of ISG15 in Sec10-overexpressing HeLa cell lines infected with SeV for the indicated time points or transfected with 5'ppp-dsRNA for 8 h. (**D**) Real-time PCR analysis of* IFNB*, *ISG15*,* CXCL10* and *CCL5* mRNA expression in Sec10-specific siRNA (siSec10) or negative control siRNA (siNC) transfected HeLa cell lines infected with SeV, MDBK cell lines with BEFV infection and A549 cell lines infected with H1N1 for 12 h, or Sec10 silencing HeLa cells transfected with 5'ppp-dsRNA for 8 h. (**E, F**) Immunoblot analysis of the protein expression of ISG15 in Sec10-specific siRNA (siSec10) or negative control siRNA (siNC) transfected HeLa cells infected with SeV for the indicated time points, and transfected with 5'ppp-dsRNA for 8 h. Data in (B, C, E and F) are representative one of three independent experiments. Data in (A and D) are presented as mean±S.D from three independent experiments, two-way ANOVA. Ns, not significant. *P<0.05, **P<0.01, and ***P<0.001.

To establish Sec10 promotes viral replication via suppressing innate immune response, we next sought to evaluate the function of Sec10 on innate immune response. By overexpressing Sec10 in HeLa cells, we found that Sec10 markedly suppressed transcription of* IFNB* and antiviral cytokines (*ISG15*, *CXCL10* and* CCL5*) induced by SeV compared to control cells (Fig. 2A). Similar results were also observed in H1N1-infected A549 cells, BEFV-infected MDBK cells that were overexpressed for Sec10 (Fig. 2A). Moreover, we demonstrated that the transcripts of *IFNB* and antiviral genes were downregulated in Sec10-overexpressing HeLa cells after 5'ppp-dsRNA treatment (Fig. 2 A), which is a synthetic ligand for the retinoic acid-inducible protein I (RIG-I). Consistently, ectopic expression of Sec10 greatly inhibited the protein expression of antiviral genes, including ISG15 in SeV-infected or 5'ppp-dsRNA treated HeLa cells (Fig. 2B, C). In contrast, knockdown of Sec10 specifically augments SeV-induced* IFNB* and antiviral cytokines (*ISG15*, *CXCL10* and *CCL5*) mRNA production (Fig. 2D). Accordingly, knockdown of Sec10 by siRNAs in H1N1-infected A549 cells, BEFV-infected MDBK cells or 5'ppp-dsRNA-transfected HeLa cells substantially potentiated the mRNA expression of *IFNB* and antiviral cytokines (Fig. 2D). Correspondingly, knockdown of Sec10 also resulted in increased protein expression of ISG15 induced by SeV infection or 5'ppp-dsRNA transfection in HeLa cells (Fig. 2E, F). Collectively, these results suggest a negative regulatory role of Sec10 in innate immune response during viral infection.

### Sec10 deficiency promotes antiviral IFN-I production after RNA virus infection in primary macrophages

**Figure 3:**
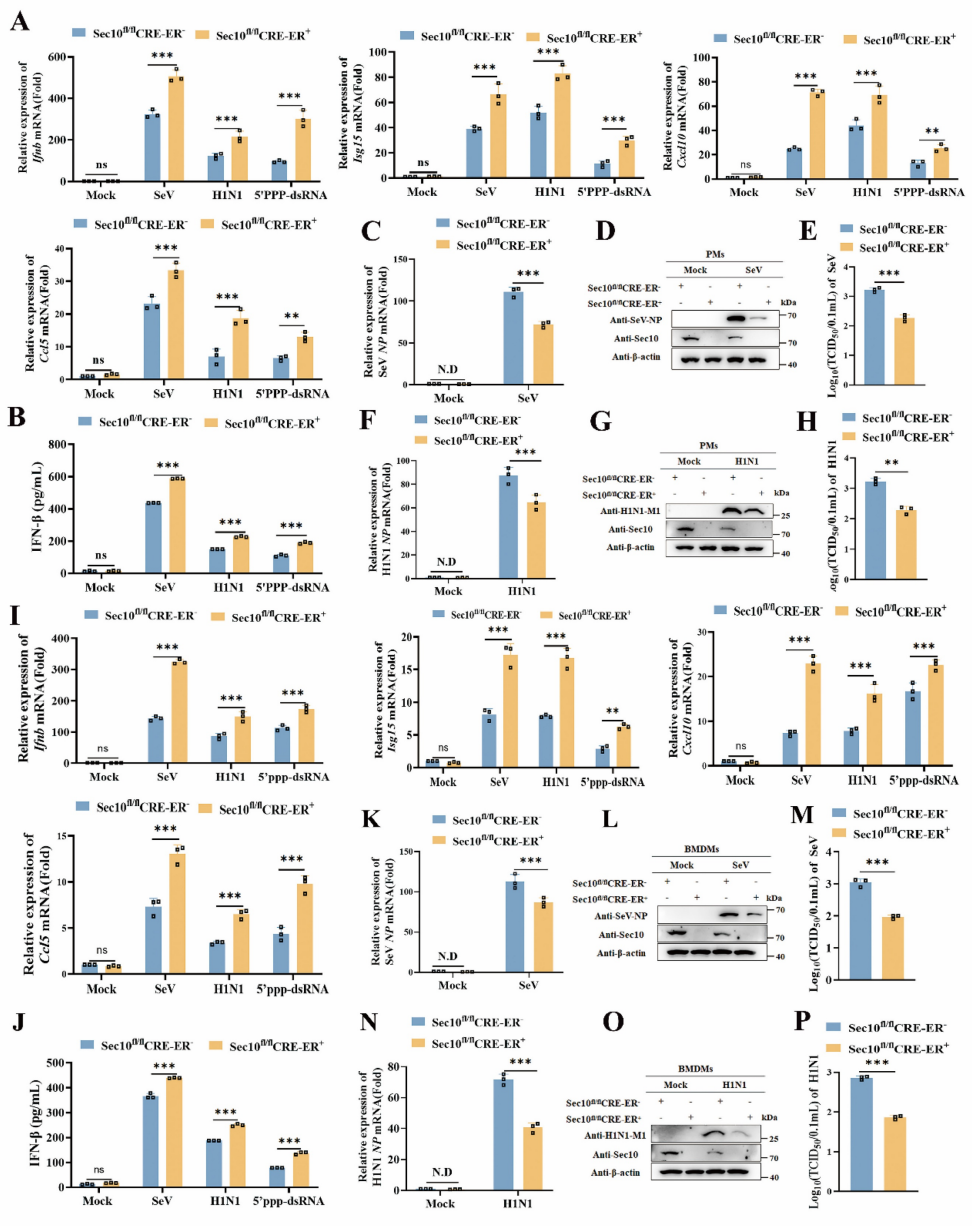
** Sec10 deficiency in primary macrophages promotes RNA virus-induced antiviral immune response.** (**A**) Real-time PCR analysis of *Ifnb, Isg15*,* Cxcl10* and* Ccl5* in WT (Sec10^fl/fl^CRE-ER-) and Sec10-KO (Sec10^fl/fl^CRE-ER +) PMs that were left untreated (Mock) or infected with SeV, H1N1 or transfected with 5'ppp-dsRNA (200 ng/mL) for 8 h. (**B**) ELISA of IFN-β protein levels in the supernatant of WT (Sec10^fl/fl^CRE-ER-) and Sec10-KO (Sec10^fl/fl^CRE-ER+) PMs infected with SeV, H1N1 or transfected with 5'ppp-dsRNA (200 ng/mL) for 8 h. (**C-H**) Real-time PCR and Western blotting analysis of the expression levels of the viral genes, proteins, and TCID_50_ assays of the viral titer in SeV (C-E) or H1N1 (F-H) infected WT (Sec10^fl/fl^CRE-ER-) and Sec10-KO (Sec10^fl/fl^CRE-ER+) PMs for 24 h, respectively. (**I**) Real-time PCR analysis of *Ifnb, Isg15*,* Cxcl10* and* Ccl5* in WT (Sec10^fl/fl^CRE-ER-) and Sec10-KO (Sec10^fl/fl^CRE-ER+) BMDMs that were left untreated (Mock) or infected with SeV, H1N1 or transfected with 5'ppp-dsRNA (200 ng/mL) for 8 h. (**J**) ELISA of IFN-β protein levels in the supernatant of WT (Sec10^fl/fl^CRE-ER-) and Sec10-KO (Sec10^fl/fl^CRE-ER+) BMDMs infected with SeV, H1N1 or transfected with 5'ppp-dsRNA (200 ng/mL) for 8 h. (**K-P**) Real-time PCR analysis and Western blotting analysis of the expression levels of the viral genes, proteins, and TCID_50_ assays of the viral titer in SeV (K-M) or H1N1 (N-P) infected WT (Sec10^fl/fl^CRE-ER-) and Sec10-KO (Sec10^fl/fl^CRE-ER+) BMDMs for 24 h. Data in (D, G, L and O) are representative one of three independent experiments. Data in (A-C, E, F, H-K, M, N and P) are presented as mean±S.D from three independent experiments, two-way ANOVA for data in (A, B, I and J), and two-tailed Student's t test for date in (C, E, F, H, K, M, N and P). N.D, not detected. Ns, not significant, **P<0.01, and ***P<0.001.

To further determine the effect of Sec10 on antiviral immune response, we extend our results to the primary macrophages in a more physiological context. We firstly generated a Sec10 knockout mouse model. Since loss of Sec10 is embryonic lethal^22^, and therefore we engineered the conditional knockout mice with the tamoxifen inducible Cre recombinase model (ERT2-CRE) (Fig.S2). Thus, primary cells including peritoneal macrophages (PMs) and bone marrow-derived macrophages (BMDMs) were isolated from wild type (Sec10^fl/fl^CRE-ER-) and Sec10 conditional knockout (Sec10^fl/fl^CRE-ER+) mice. These cells were treated with 4-hydroxytamoxifen (4OH-tamoxifen) and then treated with diverse virus infections and 5'ppp-dsRNA stimuli. The results showed that the mRNA expression of *Ifnb* and antiviral cytokines induction in PMs with Sec10 deficiency in response to SeV or H1N1 infection as well as 5'ppp-dsRNA transfection was drastically enhanced when compared with wild-type (WT) cells (Fig.3A). In addition, IFN-β protein secretion was also increased in Sec10 deficiency PMs upon RNA virus infection or 5'ppp-dsRNA stimulation compared with WT cells (Fig.3B). Accordingly, loss of Sec10 dramatically suppressed virus propagation, as reflected by decreased SeV *NP*, H1N1 *NP* mRNA level, SeV NP and H1N1 M1 protein expression and virus titers in SeV or H1N1-infected Sec10 deficient PMs (Fig. 3C-H). Consistently, Sec10 deficiency BMDMs significantly promoted *Ifnb*, *Isg15*, *Cxcl10* and *Ccl5* mRNA levels and IFN-β protein secretion in response to diverse stimuli compared to the control cells (Fig. 3I, J). Similarly, Sec10 deficiency-reduced SeV and H1N1 replication was also verified in BMDMs (Fig. 3K-P). In summary, these results demonstrate that Sec10 is indeed involved in RNA virus-triggered antiviral immune response in the primary macrophages.

### Sec10 suppresses IFN-I response by downregulating RIG-I expression at the transcriptional level

**Figure 4:**
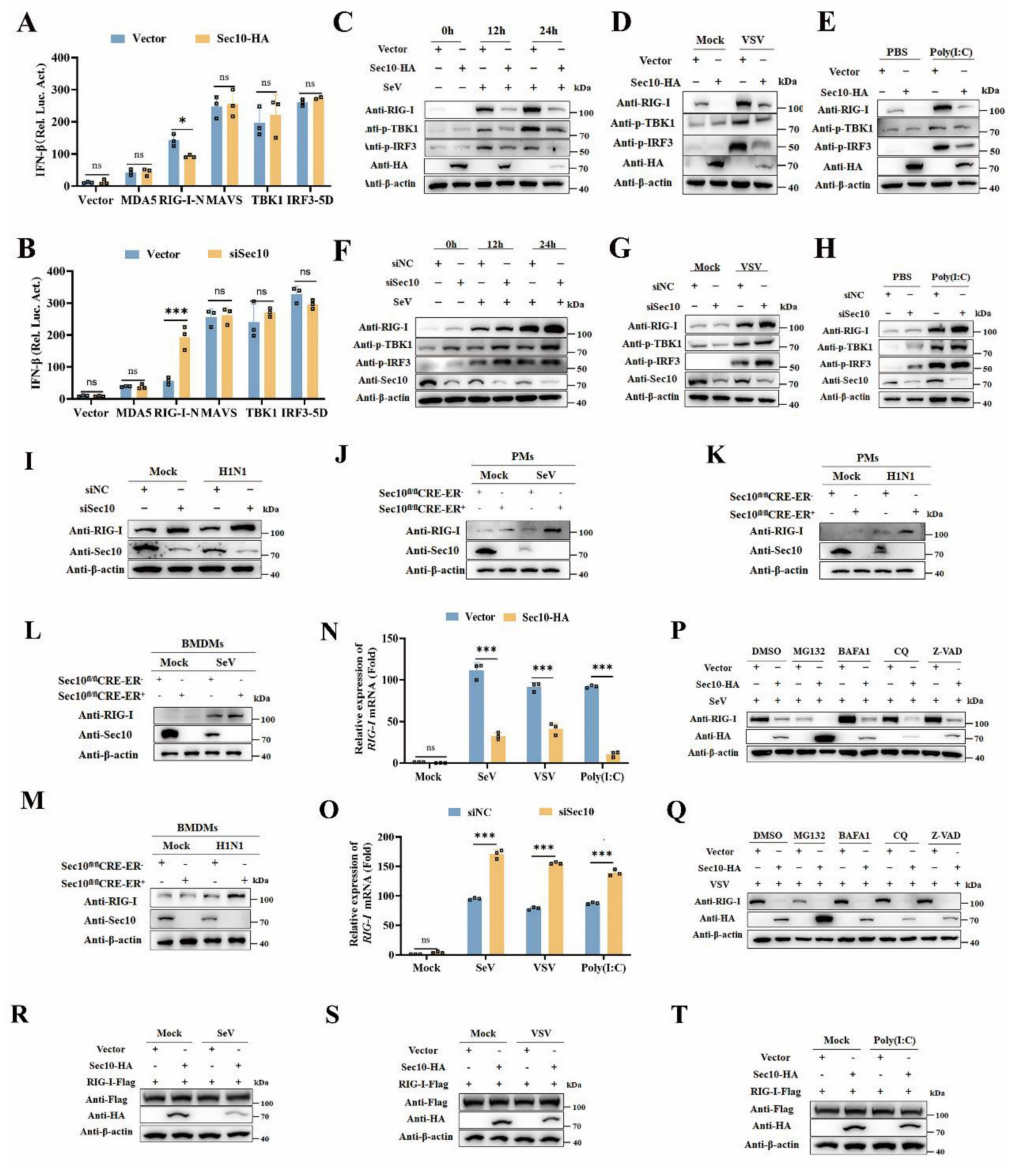
** Sec10 suppresses IFN-I response by targeting and downregulating RIG-I expression at the transcriptional level.** (**A, B**) Luciferase reporter assays of HEK293T cells transfected with IFN-β-Luc promoter plasmids, pRL-TK (Renilla luciferase reporter plasmids), the indicated expression plasmids together with Sec10-HA or empty vector (A), or Sec10-specific siRNA (siSec10) or negative control siRNA (siNC) (B) for 24 h. (**C-E**) Immunoblot analysis of RIG-I and phosphorylated TBK1 and IRF3 expression in Sec10-overexpressing HeLa cell lines infected with SeV(C), VSV (D) or transfected with low-molecular-weight (LMW) poly(I: C) (10 μg/mL) (E) for the indicated time points. (**F-H**) Immunoblot analysis of RIG-I and phosphorylated TBK1 and IRF3 expression in Sec10-specific siRNA (siSec10) or negative control siRNA (siNC) transfected HeLa cells infected with SeV(F), VSV (G) or transfected with poly(I: C) (H) for the indicated time points. (**I-M**) Immunoblot analysis of RIG-I expression in Sec10-specific siRNA (siSec10) or negative control siRNA (siNC) transfected A549 cells infected with H1N1(I), SeV or H1N1-infected Sec10-KO(Sec10^fl/fl^CRE-ER-), control (Sec10^fl/fl^CRE-ER+) PMs (J, K) and BMDMs (L, M). (**N, O**) Real-time PCR analysis of* RIG-I* mRNA expression in Sec10-overexpressing or control HeLa cell lines (N) or Sec10-specific siRNA (siSec10) or negative control siRNA (siNC) transfected HeLa cells (O) with SeV, VSV infection, or transfected with poly(I:C) (10 μg/mL) for 12 h. (**P, Q**) Western blotting analysis the protein expression of RIG-I in Sec10-overexpressing HeLa cell lines infected with SeV for 12 h, followed by treatment with DMSO, MG132, BAFA1, CQ and Z-VAD. **(R-T)** Western blotting analysis the exogenous protein expression of RIG-I in Sec10-overexpressing HeLa cell lines transfected with RIG-I-Flag plasmid and infected with SeV, VSV or transfected with poly(I: C) (10 μg/mL) for 12 h. Data in (C-M, P-T) are representative one of three independent experiments. Data in (A, B, N and O) are presented as mean±S.D from three independent experiments, two-way ANOVA. Ns, not significant, *P<0.05, and ***P<0.001.

To determine the molecular targets of Sec10 in type I IFN production, we performed luciferase reporter assays to investigate whether Sec10 could regulate the activation of IFN-β promoter induced by MDA5, RIG-I-N, MAVS, TANK binding kinase 1 (TBK1), and constitutively active IRF3-5D. As shown in Figure 4A, overexpression of Sec10 inhibited the IFN-β luciferase reporter induced by RIG-I but not by other signaling molecules above. In contrast, knockdown of Sec10 had the opposite effect (Fig. 4B), suggesting that Sec10 might function through RIG-I. Furthermore, we investigate the hallmarks of the antiviral innate immune response, i.e., phosphorylation of TBK1 and IRF3 (p-TBK1 and p-IRF3, respectively) in response to viral infection or Poly(I: C) stimulation, and found that overexpression of Sec10 suppressed the protein expression of RIG-I and reduced the levels of p-TBK1 and p-IRF3 compared to the control group upon SeV, vesicular stomatitis virus (VSV) infection or Poly(I: C) transfection (Fig. 4C-E), while knockdown of Sec10 had the opposite effect (Fig. 4F-H). This is consistent with the above finding that Sec10 can suppress innate immune response in response to RNA viruses. In line with these results, we found that RIG-I levels were also increased in H1N1-infected A549 upon Sec10 knockdown(Fig. 4I). Similar results were also obtained in Sec10 deficient-PMs or BMDMs in response to viral infection (Fig. 4J-M). All these data suggested that Sec10 suppressed IFN-I response through reduction of RIG-I level.

To further investigate molecular mechanisms behind the regulation of Sec10 on the RIG-I expression, we firstly examined the effects of Sec10 on the transcription of *RIG-I*. We found that Sec10 overexpression suppressed the transcription of *RIG-I* in response to SeV, VSV infection or Poly(I: C) transfection (Fig. 4N), whereas Sec10 knockdown had the opposite effect (Fig. 4O). Furthermore, to determine that whether protein degradation systems contribute to RIG-I protein, we treated Sec10-expressing cells with protein inhibitors including proteasome inhibitor MG132, autophagy inhibitor BafA1 and CQ, and pan caspase inhibitor Z-VAD, in response to viral infection. Notably, the treatment with protein inhibitors failed to restore the expression of RIG-I protein (Fig. 4P and 4Q). Consistent with these results, overexpression of Sec10 had no effect on exogenous expression of RIG-I upon SeV, VSV infection or Poly(I: C) transfection (Fig. 4R-T), excluding the possibility that the reduced RIG-I expression was caused by exacerbated protein degradation. Collectively, these data suggest that Sec10 suppresses the RNA virus-triggered IFN-I response by downregulating RIG-I at the transcriptional level.

### Sec10 inhibits ATF4-mediated RIG-I transcriptional regulation

**Figure 5:**
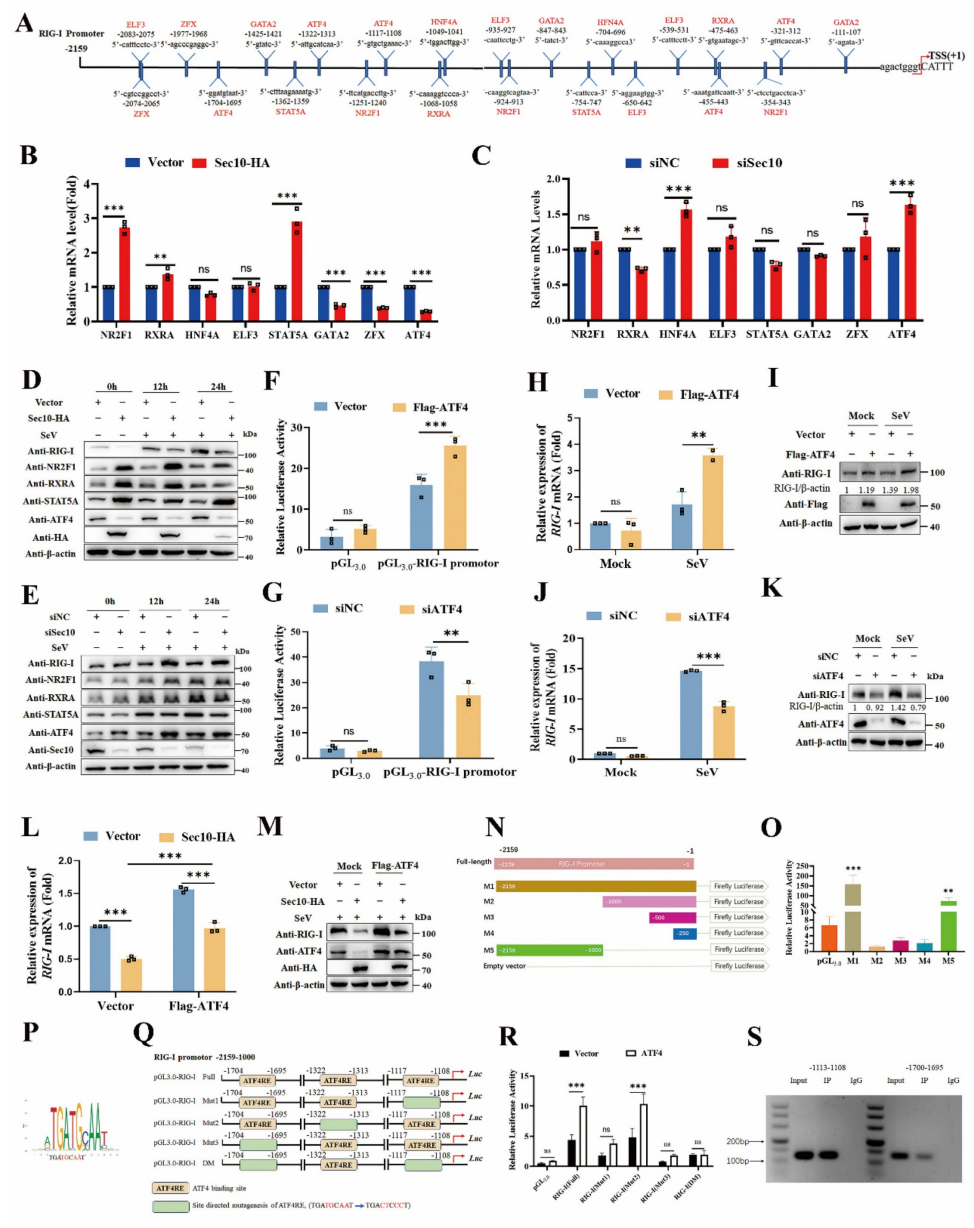
** Sec10 inhibits ATF4-mediated RIG-I transcriptional expression.** (**A**) Schematic diagram of the differential expression transcription factors that bind the RIG-I promoter sequence. (**B, C**) Real-time PCR analysis of the candidate transcription factors mRNA level in Sec10-overexpressing or control HeLa cell lines (B) or Sec10-specific siRNA (siSec10) or negative control siRNA (siNC) transfected HeLa cells (C) with SeV infection for 12 h. (**D, E**) Immunoblotting analysis of the indicated protein expression in Sec10-overexpressing or control HeLa cell lines (D) or Sec10-specific siRNA (siSec10) or negative control siRNA (siNC) transfected HeLa cells (E) with SeV infection at indicated time points. (**F, G**) Luciferase activity in HEK293T cells transfected with Flag-ATF4 or control vector (F), ATF4-specific siRNA (siATF4) or scrambled siRNA (siNC) (G) together with pRL-TK, RIG-I-Luc promoter plasmids, or Luc promoter vector. (**H-K**) Real-time PCR and immunoblotting analysis of RIG-I mRNA and protein expression in HeLa cells transfected with Flag-ATF4 or control vector (H, I) or ATF4-specific siRNA (siATF4) or negative control siRNA (siNC) (J, K) and infected with SeV for 12 h. (**L, M**) Real-time PCR and immunoblotting analysis of RIG-I mRNA and protein expression in Sec10-overexpressing or control HeLa cell lines transfected with Flag-ATF4 or empty vector for 24 h, followed by infection with SeV for 12 h. (**N**, **O**) Schematic representation of the constructed luciferase reporter by using different truncated promoter sequences of RIG-I genes, which were then used to test the transcriptional activity of RIG-I. (**P**) The potential binding sites of ATF4 in the promoter sites of RIG-I. (**Q**) Schematic showing three conserved ATF4-binding motifs located in the promoter region of RIG-I gene between -2,159 and -1,000. (**R**) Luciferase reporter assays were performed with HEK293T cells transfected with pRL-TK, wild type RIG-I-Luc promoter plasmids (Full), or mutant RIG-I-Luc promoter mutants; together with the vector or expression vector encoding ATF4. (**S**) ChIP analysis of the binding activity of ATF4 in the RIG-I promoter region *in vivo*. Antibody to ATF4 or rabbit IgG was used to precipitate chromatin-bound ATF4. Normal rabbit IgG was used as a negative control. Data in (D, E, I, K, M and S) are representative one of three independent experiments. Data in (B, C, F-H, J, L, O and R) are presented as mean±S.D from three independent experiments, two-way ANOVA. Ns, not significant, **P<0.01, and ***P<0.001.

To pinpoint the mechanism of Sec10 action on RIG-I transcriptional expression, we conducted bioinformatics prediction analysis of transcription factors (TFs) that may bind to the RIG-I promoter region based on the sequence of the promoter region of RIG-I (Fig.5A). And 8 candidate transcription factors have been selected as potential regulators of the RIG-I transcriptional expression. We then further determined the expression of these potential transcription factors upon overexpression/knockdown of Sec10. As shown in Figure 5B and 5C, overexpression of Sec10 promoted the mRNA level of NR2F1, retinoid X receptor alpha (RXRA), and signal transducer and activator of transcription 5A (STAT5A) and suppressed GATA binding protein 2 (GATA2), Zinc finger protein X-linked (ZFX) and ATF4 mRNA level. However, knockdown of Sec10 had the opposite effect on RXRA and ATF4 but not NR2F1, STAT5A, GATA2 and ZFX. Also, knockdown of Sec10 significantly increased hepatocyte nuclear factor 4 alpha (HNF4A) mRNA levels, whereas it had no effect upon Sec10 overexpression. Furthermore, overexpression of Sec10 increased the protein expression of NR2F1, RXRA and STAT5A and decreased ATF4 protein expression (Fig.5D), whereas knocking down Sec10 only significantly promoted the protein expression of ATF4 (Fig.5E). Therefore, we speculate that Sec10 may inhibit the effect of ATF4 on RIG-I transcriptional activation. As expected, overexpression of ATF4 enhanced the promoter activity of RIG-I, whereas silencing of ATF4 suppressed this effect (Fig.5F, G). Moreover, overexpression of ATF4 significantly increased RIG-I mRNA and protein expression as compared to the vector control during SeV infection (Fig.5H, I). Conversely, silencing of ATF4 exhibited an opposite effect (Fig.5J, K), indicating that ATF4 promotes the transcriptional level of RIG-I. Notably, the inhibitory effect on RIG-I mRNA and protein expression by Sec10-expressing was significantly weakened as compared with that of control cells after overexpression of ATF4 (Fig.5L, M). Since RIG-I is also a well-known interferon-stimulated gene, interferon receptor knockout does not alter Sec10-mediated transcriptional repression of RIG-I (Fig. S3), these results rule out the possibility that Sec10 affects IFN-induced expression of RIG-I. Collectively, the above results suggested that Sec10 regulates RIG-I transcription via downregulation of ATF4.

To confirm the direct transcriptional facilitation function of ATF4 on *RIG-I* level, we generated luciferase reporter plasmids by introducing different RIG-I promoter fragments into a pGL_3.0_-basic plasmid and found that the promoter region between -2,159 and -1 contributed to the basal expression of RIG-I (Fig. 5N). In addition, we also determined that the promoter region between -2,159 and -1,000 effectively promoted RIG-I transcription, while the promoter region between -1,000 and -1 suppressed RIG-I transcription (Fig. 5O). Notably, the position weight matrices (PWMs) for ATF4 binding at specific sites within the RIG-I promoter sequence were obtained from the JASPAR CORE database (Fig. 5P). As expected, three conserved ATF4-binding motifs were found in the promoter region between -2,159 and -1,000 (Fig. 5Q). Subsequently, a series of single or both binding sites mutants of RIG-I promoter (named Mut1-Mut3, and DM) were constructed (Fig. 5Q). The luciferase reporter analysis demonstrated that ATF4 significantly increased the activity of the undivided RIG-I and single binding sites promoter mutant (Mut2). By contrast, ATF4 failed to activate RIG-I transcription when four bases of the ATF4-binding motif in single binding sites#1(Mut1), single binding sites#3 (Mut3) or both binding sites#1 and #3 were mutated (DM), compared to the full-length promoter (Fig. 5R). Moreover, the chromatin immunoprecipitation (ChIP) combined with PCR experiment also confirmed that abundant ATF4 binds to the *RIG-I* promoter region at -1113 to -1108 and -1700 to -1695 (Fig. 5S). Together, these data demonstrate that Sec10 suppresses ATF4-mediated *RIG-I* transcription regulation during RNA virus infection.

### Sec10 inhibits antiviral immune response via NRF2/ATF4/RIG-I axis

**Figure 6:**
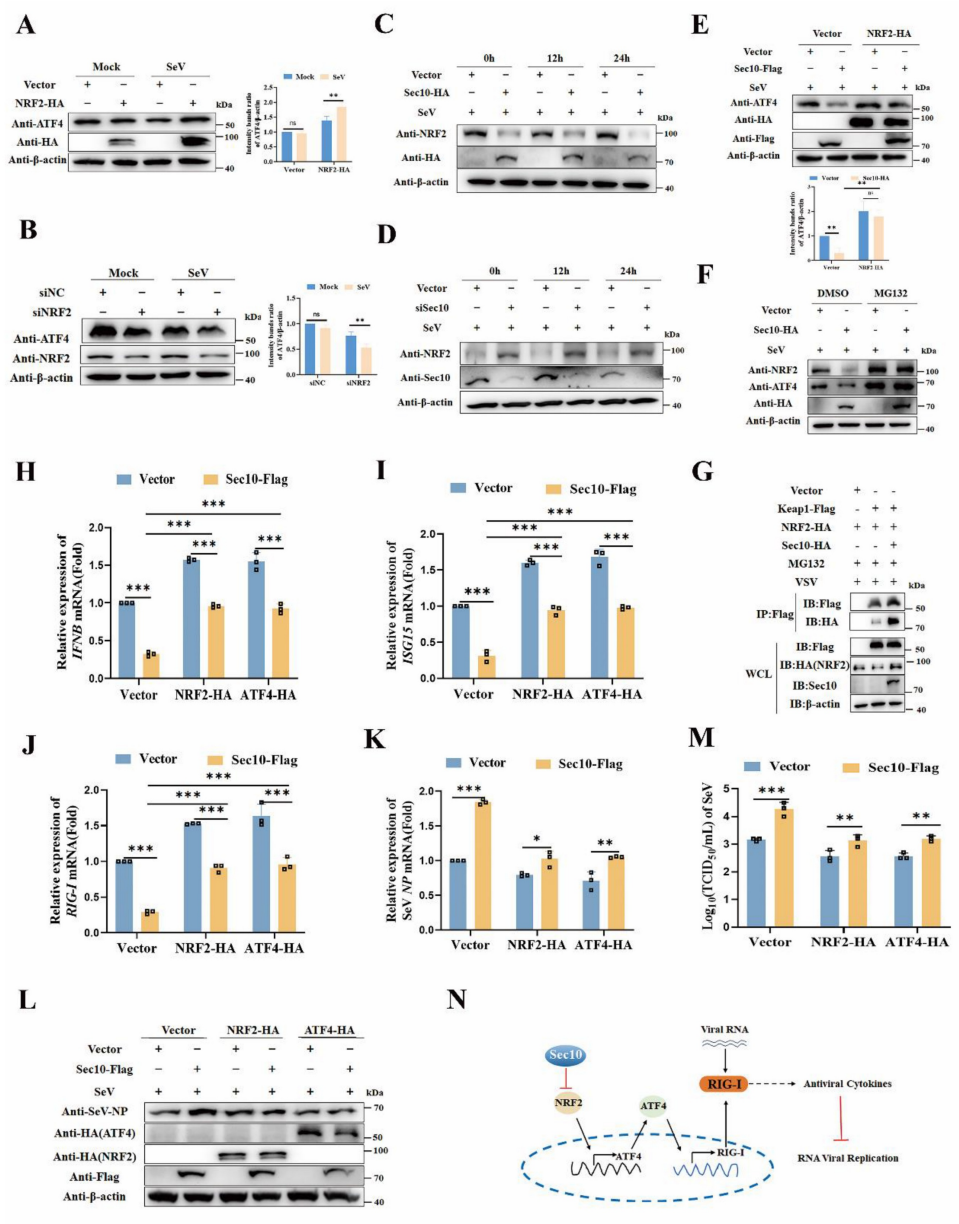
** Sec10 inhibits antiviral immune response via NRF2/ATF4/RIG-I axis.** (**A, B**) Immunoblotting analysis of ATF4 protein in HeLa cells transfected with NRF2-HA or empty vector for 24 h, or NRF2-specific siRNA (siNRF2) or negative control siRNA (siNC), followed by left uninfected (Mock) or infected with SeV for 12 h. (**C, D**) Immunoblotting analysis of NRF2 protein in Sec10-overexpressing or control HeLa cell lines (C) or Sec10-specific siRNA (siSec10) or negative control siRNA (siNC) transfected HeLa cells (D) with SeV infection for indicated time points. (**E**) Immunoblotting analysis of ATF4 protein in Sec10-overexpressing or control HeLa cell lines transfected with NRF2-HA or empty vector, and infected with SeV for 24 h. (**F**) Immunoblotting analysis of NRF2 protein in SeV-infected Sec10-overexpressing or control HeLa cell lines after treatment with MG132. (**G**) Co-immunoprecipitation (co-IP; with anti-FLAG) and immunoblotting analysis using protein lysates of HEK293T cells transfected with indicated plasmids. WCL, whole cell lysates. (**H-K**) Real-time PCR analysis of *IFNB*, *ISG15* and* RIG-I* and viral burden of SeV *NP* mRNA expression levels in Sec10-overexpressing or control HeLa cell lines transfected with NRF2-HA, ATF4-HA or empty vector, and infected with SeV for 12 h. (**L, M**) Immunoblotting analysis of viral NP protein and TCID_50_ analysis of the viral titer in SeV-infected Sec10-overexpressing or control HeLa cell lines transfected with NRF2-HA, ATF4-HA or empty vector. **(N)** Schematic presentation of Sec10 inhibits antiviral immune response via NRF2/ATF4/RIG-I axis. Data in (A-G, L) are representative one of three independent experiments. Data in (H-K, M) are represented as means±S.D from three independent experiments, two-way ANOVA. *P < 0.05, **P < 0.01, ***P < 0.001.

Next, we sought to investigate how Sec10 modulates ATF4 expression upon virus infection. NRF2 is important to ATF4 activation^23^, making it an attractive candidate to act in a NRF2-ATF4 pathway that leads to increased RIG-I expression. Therefore, we tested the role of Sec10 in the NRF2-ATF4 signaling pathway. Our findings indicate that overexpression of NRF2 led to an increase in ATF4 expression (Fig. 6A), in contrast, silencing NRF2 resulted in the opposite effect (Fig. 6B). Furthermore, we observed a time-dependent decrease in NRF2 protein levels following SeV infection when Sec10 was overexpressed (Fig. 6C), while knockdown of Sec10 obtained the opposite effect (Fig. 6D), suggesting that Sec10 suppressed the NRF2 expression. Notably, after overexpression of NRF2, the expression ability of ATF4 inhibited by Sec10 was significantly reduced compared to the empty vector control group (Fig. 6E). These data establish the relationship that Sec10 downregulates ATF4 through NRF2. Furthermore, the overexpression of Sec10 led to a downregulation of NRF2, which, following treatment with MG132, led to a recovery in the expression of NRF2. Similarly, MG132 treatment significantly alleviated the inhibitory effect of Sec10 on ATF4 (Fig. 6F), indicating that Sec10 may degrade NRF2 through the ubiquitin-proteasome pathway, thereby suppressing ATF4 expression. Keap1 acts as an endogenous inhibitor of NRF2, sequestering and targeting NRF2 for proteasomal degradation under basal conditions^24^, ^25^. Subsequently, we investigated whether Sec10 inhibits the expression of NRF2 through Keap1-mediated proteasomal degradation. As expected, the immunoprecipitation analysis showed that overexpression of Sec10 promoted the binding of Keap1 and NRF2 (Fig. 6G). After silencing Keap1, the downregulated NRF2 caused by Sec10 was restored (Fig. S4), indicating that Sec10 reduces NRF2 expression through Keap1.

To further verify that Sec10 participates in antiviral innate immunity by suppressing the NRF2/ATF4/RIG-I signaling axis, we determined the antiviral innate immune response following NRF2/ATF4 expression. Consistent with our expectations, overexpression of NRF2 or ATF4 in Sec10-expression cells significantly attenuated the Sec10-downregulated *IFNB* and* ISG15* mRNA expression compared with that in the control group upon virus stimulation (Fig. 6H and I). Consistently, the overexpression of NRF2 or ATF4 disrupted the Sec10-reduced *RIG-I* transcription (Fig. 6J). Accordingly, the overexpression of NRF2 or ATF4 in Sec10-expression cells significantly diminished the Sec10-enhanced RNA replication, as evidenced by reduced mRNA and protein expression of the SeV NP gene, and reduced virus titers of SeV (Fig. 6K-M). All in all, these findings confirm the mechanism by which Sec10 suppresses antiviral immunity and promotes RNA virus replication via the NRF2-ATF4-RIG-I signaling axis (Fig. 6N).

### Sec10 deficiency alleviates RNA virus infection in mice

**Figure 7:**
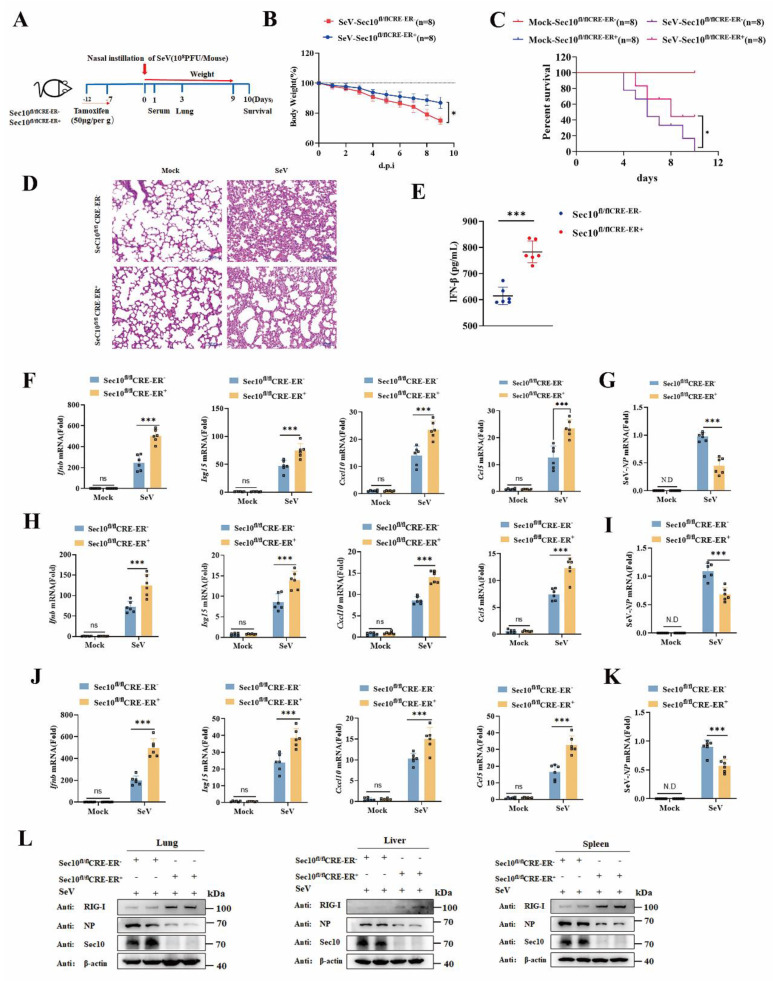
** Loss of Sec10 strengthens IFN-I response against SeV infection *in vivo*.** (**A**) Schematic presentation of the experimental workflow. The Sec10^fl/fl^CRE-ER-, and Sec10^fl/fl^CRE-ER+ mice were inoculated with SeV (10^8^PFU per mouse). (**B**) The body weights of Sec10^fl/fl^CRE-ER- and Sec10^fl/fl^CRE-ER+ mice (n=8 mice per group) inoculated with SeV (10^8^PFU per mouse) were recorded. (**C**) Kaplan-Meier survival curves of different groups of mice (Sec10^fl/fl^CRE-ER- and Sec10^fl/fl^CRE-ER+ mice) in response to SeV (10^8^PFU per mouse) or mock infection, (n = 8 mice per group). (**D**) Representative H&E-stained images of lung sections from Sec10^fl/fl^CRE-ER- and Sec10^fl/fl^CRE-ER+ mice with or without SeV (10^8^PFU per mouse) infection for 3 d. Scale bars, 100 µm. (**E**) ELISA for IFN-β in serum of Sec10^fl/fl^CRE-ER- and Sec10^fl/fl^CRE-ER+ mice infected with SeV (10^8^PFU per mouse) for 24 h. (**F-K**) Real-time PCR analysis of the mRNA expression of *Ifnb* and ISGs (*Isg15, Cxcl10* and* Ccl5*) and the viral load of NP gene in the lung (F, I), liver (G, J) and spleen (H, K) from Sec10^fl/fl^CRE-ER- and Sec10^fl/fl^CRE-ER+ mice after SeV infection. (**L**) Immunoblotting analysis of RIG-I and viral gene expression in the lung, liver, and spleen tissues from the Sec10^fl/fl^CRE-ER- and Sec10^fl/fl^CRE-ER+ mice infected with SeV (10^8^PFU per mouse). Data in (D, L) are representative of three independent experiments. Data in (B, C, E-K) are presented as mean±S.D., n=8 for (B, C), n=6 for (E-K) biologically independent experiments. Statistical significance was determined using two-tailed Student's t tests in (B, E, G, I, K) or two-way ANOVA in (F, H, J). Ns, not significant, *P<0.05, and ***P<0.001.

To investigate the function of Sec10 in the antiviral innate immune response *in vivo*, wild type (WT, Sec10^fl/fl^CRE-ER-) and Sec10 conditional knockout (Sec10^fl/fl^CRE-ER+) mice were induced with tamoxifen to globally knock out Sec10, and then infected intranasally with SeV at postinduction day 7 (Fig.7A). As expected, the loss body weight of the SeV-infected Sec10 knockout mice was lower than that of WT mice (Fig. 7B). The survival rate and histological analysis showed that Sec10 deficiency significantly increased the SeV-induced mouse survival rate and alleviated immune cell infiltration and lung tissue impairment (Fig.7C and 7D), suggesting that Sec10 deficiency reduces the susceptibility to SeV infection. In addition, the production of IFN-β cytokines in the serum was much higher in the SeV-infected Sec10 mice than in the WT controls (Fig. 7E). Consistently, the mRNA expression of *Ifnb*, *Isg15*,* Cxcl10* and *Ccl5* induced by SeV infection in the lungs, livers and spleens of the Sec10 knockout mice was significantly augmented in comparison to that of WT mice (Fig. 7F, H, J). Accordingly, loss of Sec10 markedly suppressed virus propagation *in vivo* after SeV infection, as reflected by increased SeV expression at both mRNA (Fig. 7G, I, K) and protein levels (Fig. 7L) in the lungs, livers and spleens of SeV-infected Sec10-deficient mice compared with WT control mice. In agreement with this pattern, a higher protein expression of RIG-I was observed in the lungs, livers and spleens of Sec10-deficient mice than in the same tissues of WT counterparts (Fig. 7L). Collectively, these data suggest that Sec10 is critical for attenuating antiviral immunity against SeV infection in mice.

## Discussion

The RLR signaling axis has been identified for its essential role in antiviral defense and IFN-I induction during RNA viral infection^11^, ^26^. In this study, for the first time, we identified Sec10 as a specific negative regulator of antiviral immunity, targeting the reduction of RIG-I expression at the transcriptional level during RNA viral infection. Importantly, deficiency of Sec10 resulted in increased RIG-I expression, enhanced the production of type I interferons and antiviral cytokines in response to RNA virus infections, leading to enhanced innate immune responses, decreased viral load and lower morbidity *in vivo*. This is consistent with previous observations that individual subunits of the exocyst can affect the proliferation of viruses or other organisms by regulating innate immune responses. For instance, Sec3 could negatively regulate immune response to promote Singapore grouper iridovirus infection^27^. Sec5 enhances antifungal innate immune responses by potentiating the activation of TBK1 and IRF3^28^. Additionally, Exo70 promotes Dengue virus egression/secretion without influencing viral transcription and translation^29^. Virus infection induces the assembly of Exo84- protein kinase R (PKR) - macrophage stimulating 1 (MST1) and Sec5-TBK1-mTOR complexes to integrate Hippo and mTOR signaling to promote virus detection^30^. This is an indication that individual subunits of the exocyst display differential regulation, each playing a distinct role and involving different molecular mechanisms in the control of viral replication. Furthermore, we observed that the protein level of Sec10 was increased in the early phase of viral infection, followed by significant downregulation in different types of cells during the later stages of various RNA virus infection (Fig. S1). However, its expression was restored upon MG132 treatment (Fig. 4P, Q), implying that Sec10, as an important antiviral immunity response regulatory factor, is downregulated upon viral infection. Specifically, we found that Sec10 was downregulated during the later stages of viral infection through the SMAD specific E3 ubiquitin protein ligase 1 (SMURF1)-mediated ubiquitin-proteasome pathway (Fig. S5). Based on this, we speculate that in the early phase of viral infection, virus upregulates Sec10 to inhibit the host antiviral innate immune response and promote viral replication. Subsequently, the host counteracts this by activating the ubiquitin-proteasome pathway to downregulate Sec10, thereby enhancing its defensive capacity to combat viral infection to avoid excessive damage.

There is substantial evidence indicating that post-translational modifications of RIG-I are crucial for regulating the induction of type I interferon (IFN) in response to viruses^31^, ^32^. Furthermore, gene expression is under tight control of transcription factors that bind to unique DNA enhancer/repressor elements. However, the mediators regulating the transcriptional expression of RIG-I and the specific mechanisms by which RIG-I regulation at the mRNA level is accomplished during viral infection remain poorly understood. Herein, our data establishes a crucial role for Sec10 in the negative regulation of RIG-I transcriptional level. Bioinformatics analysis combined with luciferase assay and ChIP assay illuminated that ATF4 binds to the RIG-I promoter at the -1117 to -1108, -1744 to -1695 regions. Overexpression/knockdown of ATF4 enhanced/suppressed the *RIG-I* mRNA expression levels, indicating that ATF4 acts as a positive transcriptional regulator of RIG-I. ATF4 is a basic region leucine zipper transcription factor that plays pivotal roles in physiological responses to stresses including hypoxia, endoplasmic reticulum stress, amino acid deprivation, oxidation, and mitochondrial stress^33^, ^34^. ATF4 has been described to have proviral functions, such as directly controlling cellular transcription to promote human immunodeficiency virus (HIV-1), human herpes virus 8, and murine cytomegalovirus infections^35^-^37^. Previous studies have found that ER stress induced by HIV-1 infections could activate ATF4, which then binds to the TLR2 promoter to promote its transcriptional expression in infected cells^38^. ATF4 mediates TLR4-triggered cytokine production activated by lipopolysaccharide (LPS)^39^. ATF4 has also been shown to be involved in the TLR-mediated innate antifungal immune response^40^. In addition, ATF4, whose expression is induced by viral infections and various stresses, binds to and negatively regulates IRF7 expression, although IRF7 upregulates ATF4 activity and expression^41^. In this study, we identified that ATF4 functions as a novel positive transcriptional regulator of RLR signaling pathway during RNA infection. Moreover, co-expressing Sec10 and ATF4 conferred partial restoration of RIG-I expression compared to the control group, enhancing SeV-induced IFN signaling response. These data suggest that Sec10 negatively regulates the RIG-I transcriptional expression via downregulation of ATF4. Therefore, identification of novel transcription targets of ATF4 during viral infection would contribute to the understanding of innate immune networks and help to identify novel therapeutic targets.

While eIF2α phosphorylation is required for ATF4 translation^42^, Sec10 does not inhibit ER stress-induced ATF4 expression during viral infection (Fig. S6), little is known about the transcriptional regulation of ATF4. Our study demonstrated that Sec10 downregulated ATF4 mRNA levels via NRF2-dependent mechanisms. NRF2 is a redox sensitive bZIP transcription factor that mediates adaptive responses and regulates the expression of antioxidant and detoxification enzymes^43^. Additionally, NRF2 has been implicated in repressing antiviral cytosolic sensing by decreasing the mRNA stability of the adaptor protein STING^44^. However, the exact mechanisms by which NRF2 affects cytosolic nucleic acid sensing in the context of RNA are not yet fully understood. Although NRF2 may play a role in activating the ATF4 promoter, as it binds to the ARE site within the ATF4 promoter^23^, it is possible that Sec10 deactivates the NRF2-ATF4 signaling cascade to suppress *RIG-I* mRNA expression, and thereby impedes the RLR-dependent innate immune response. Furthermore, it is supported by reports of NRF2-mediated ATF4 induction during oxidative stress^45^. In addition, Sec10 has been shown to protect epithelial barrier integrity and enhance recovery from oxidative stress in kidney epithelial cells^21^. These data raise the possibility that a cross-talk between the Sec10-NRF2-ATF4 signaling pathways and innate immune response. Regardless, future studies are required to address how changes in the NRF2-ATF4-dependent network may influence various biological processes through the modulation of the same molecules.

Interestingly, we observed that overexpression of Sec10 downregulated NRF2 protein expression. While Keap1 is the primary regulator of NRF2 stability through the CUL3-based E3 ubiquitin ligase complex, other E3 ligases, such as hydroxymethylglutaryl reductase 1(HRD1) and beta-transducin repeat containing E3 ubiquitin protein ligase (β-TrCP), can also target NRF2 for degradation^46^. Herein, we demonstrate for the first time that Sec10 may play a role in controlling NRF2 protein levels through these E3 ligases, resulting in reduced protein expression of NRF2 and translocated to the nucleus, inhibiting the ATF4-activated RIG-I, a hallmark of RLR-mediated innate immune response.

In summary, we have identified a novel function of Sec10 in contributing to RNA viral infection and decreasing the innate immune response via the inhibition of RIG-I at the transcriptional level both *in vitro* and* in vivo*. Importantly, we have uncovered the molecular details of how Sec10 deregulates NRF2 to inhibit ATF4, leading to transcriptional repression of the key gene *RIG-I*, thereby impairing the *RIG-I*-mediated antiviral immune response. Consequently, we have defined the molecular bases of how Sec10 controls antiviral immunity via the NRF2-ATF4-RIG-I signal axis.

In this study, we identified a new function of Sec10 in innate immunity against RNA viruses. However, further research is required to determine whether Sec10 plays a role in DNA virus-induced antiviral innate immune responses, and directly interferes with RIG-I signaling occur through inhibition of RIG-I activation by viral RNA and/or disruption of the activated RIG-I-MAVS interaction. Furthermore, Sec10 may play a role in controlling NRF2 protein levels through the ubiquitin-proteasome degradation. However, we did not find out the mechanism by which Sec10 downregulates NRF2 protein, the precise mechanism(s) need further investigation. Notably, the Sec10 expression levels were downregulated through the SMURF1-mediated ubiquitin-proteasome pathway in virus-infected cells, however, the precise mechanism underlying suppression and its functional implications in viral pathogenesis remain to be fully elucidated.

## Material and Methods

### Cell culture

HeLa cells (ATCC, CCL-2), HEK293T cells (ATCC, CRL-1537), A549 cells (ATCC, CCL-185) and MDBK cells (ATCC, CCL-22) were purchased from American Type Culture Collection and stored in our laboratory. Peritoneal macrophages (PMs) were harvested from 7-8-week-old Sec10^fl/fl^CRE-ER- and Sec10^fl/fl^CRE-ER+ mice 4 days post the intraperitoneal injection of starch solution (Sigma Aldrich, 232-679-6) in sterile PBS. Bone marrow-derived macrophages (BMDMs) were isolated from the femurs and tibias of 7-8-week-old Sec10^fl/fl^CRE-ER- and Sec10^fl/fl^CRE-ER+ mice. All the cells were maintained in Dulbecco's Modified Eagle's Medium (DMEM) supplemented with 10% fetal bovine serum (FBS) and 100 μg/mL antibiotics (100 U/mL penicillin and 100 μg/mL streptomycin) in a humidified incubator with 5% CO_2_ at 37°C.

### Generation of Sec10 conditional knockout mice

C57BL/6-Sec10^fl/fl^ mice were obtained from Cyagen Biosciences, which were generated by CRISPR/Cas9-mediated genome editing. Two loxP sites flank the seventh to tenth exons of the mouse Sec10 gene. Cre-ER mice (B6.129-Gt (ROSA) 26 Sortm1(Cre/ERT2) Tyj) (hereafter referred to as CRE-ER) were purchased from the GemPharmatech Co. ltd. Sec10^fl/fl^ mice were crossed with CRE-ER mice to generate Sec10^fl/fl^CRE-ER+/- mice. After crossing, the Sec10^fl/fl^CRE-ER- and Sec10^fl/fl^CRE-ER+ littermates were selected and used for further assays. Littermates who lacked the CRE gene (Sec10^fl/fl^CRE-ER-) were used as controls. The genotyping primers are listed in Supplementary Table 1.

To induce global conditional knockout of Sec10, 7-8-week-old Sec10^fl/fl^CRE-ER- and Sec10^fl/fl^CRE-ER+ mice were injected intraperitoneally with tamoxifen (50 μg/g body weight, dissolved in corn oil) for five consecutive days. After 7 days without treatment, mice were either euthanized to test the knockout efficiency or infected with SeV. Sec10^fl/fl^CRE-ER- and Sec10^fl/fl^CRE-ER+ mice used in the experiments were randomly grouped. All mice were housed under specific pathogen-free conditions at the Laboratory Animal Center of Shandong Normal University, and all animal studies conformed to the Institutional Animal Care and Use Committee of Shandong Normal University.

Peritoneal macrophages (PMs) were harvested from 7-8-week-old Sec10^fl/fl^CRE-ER- and Sec10^fl/fl^CRE-ER+ mice 4 days post the intraperitoneal injection of starch solution in sterile PBS. Bone marrow-derived macrophages (BMDMs) were isolated from the femurs and tibias of 7-8-week-old Sec10^fl/fl^CRE-ER- and Sec10^fl/fl^CRE-ER+ mice. Interferon alpha and beta receptor subunit 1 (IFNAR1)-knockout (IFNAR1-KO), SMURF1 knockdown (shSMURF1) and STIP1 homology and U-Box containing protein 1 (STUB1) knockout (STUB1-KO) HeLa cells were stored in our laboratory^47^. All the cells were maintained in Dulbecco's Modified Eagle's Medium (DMEM) supplemented with 10% fetal bovine serum (FBS) and 100 μg/mL antibiotics (100 U/mL penicillin and 100 μg/mL streptomycin) in a humidified incubator with 5% CO_2_ at 37°C.

To knock out Sec10, PMs and BMDMs from Sec10^fl/fl^CRE-ER- and Sec10^fl/fl^CRE-ER+ mice were treated with 4-hydroxytamoxifen (4OH-tamoxifen) (1 μM) for three days. Cells were reseeded into culture dishes or plated in 4-hydroxytamoxifen-free medium and allowed to rest for 24 h. BMDMs were stimulated with 20 ng/mL of macrophage colony stimulating factor (GM-CSF) for 7 days followed by infection with SeV, H1N1 or transfection with 5'ppp-dsRNA.

### Plasmids construction and small interfering RNAs (siRNAs)

Homo sapiens Sec10 coding sequence (GenBank accession no. NM_006544.4) or bovine Sec10 coding sequence (GenBank accession no. NM_001192711.2) was amplified by PCR and cloned into the pLVX-IRES-Puro vector (Clontech Laboratories, Mountain View, CA). RIG-I and its mutants promoter luciferase reporters were constructed by using the QuickChange Lightning Site-directed Mutagenesis kit following manufacturer's protocol. The constructs generated in this study are listed in Supplementary Table 1. Plasmids for ATF4 and NRF2 were cloned into the pcDNA_3.1_ vector for transient expression. The specified PCR products were amplified with the primer pairs and subsequently cloned and inserted into different vectors. All the specific sequences of the primers that were used are shown in Supplementary Table 1. All constructs were subjected to sequencing analysis for confirmation.

Plasmids Flag-MDA5, RIG-I-N (the constitutively active N-terminal domains of RIG-I), MAVS, TBK1, IRF3(5D) (a constitutively active IRF3), pRL-TK and pGL3-IFN-β-Luc used in this study have been described^48^. Chemically synthesized 21-nucleotide siRNA duplexes for Sec10, ATF4, and NRF2 were designed and synthesized by Gene Pharma Company (Shanghai, China). All siRNA sequences used for knockdown are listed in Supplementary Table 1.

### Generation of stable cell lines

To stabilize the expression of Sec10 in HeLa and MDBK cells, we used a lentiviral packaging system (Clontech Laboratories, Mountain View, CA) according to protocols as described in our previous studies^49^. Briefly, HEK293T cells were transfected with indicated plasmids to obtain lentivirus. Then, HeLa or MDBK cells were infected with lentivirus, and Puromycin selection was performed for 48 h postinfection. Subsequently, single colonies were picked and verified by immunoblotting analysis.

### RNA extraction and real-time quantitative PCR (RT-qPCR)

Total RNA was isolated using total RNA isolation kit and quantified with a NanoDrop 2000^TM^ (Thermo Fisher Scientific, Wilmington, DE, USA). RNA was followed by reverse-transcribed with the SuperScript III first-strand synthesis kit according to the manufacturer's protocols. RT-qPCR was performed using SYBR^TM^ Green Master Mix Kit and a LightCycler®480 II system (Roche) for determination of mRNA expression. β-actin was used for the normalization. And induction fold was determined with the 2^-ΔΔCt^ method. The primers used in the study are listed in Supplementary Table 1.

### Immunoprecipitation and Western blotting

Cells were collected and lysed with ice-cold lysis buffer supplemented with inhibitors for proteases and phosphatases. The proteins were boiled and loaded on SDS-PAGE gels and transferred to polyvinylidene fluoride (PVDF) membranes (LABSELECT, TM-PVDF-R-45), and blocked with 5% non-fat milk in Tris-buffered saline with 0.1% Tween® 20 detergent (TBST). The blots were incubated with the indicated primary antibodies at 4°C overnight. The next day, the blots were washed with TBST and incubated with the suitable secondary antibody at room temperature for 2 h. The blots were then visualized on an imaging system (Tanon Science & Technology Co., Ltd., Shanghai, China) with an enhanced Chemiluminescence Detection Kit for HRP detection.

### Enzyme-linked immunosorbent assay (ELISA)

The concentrations of IFN-β in culture supernatants and mouse serum were measured by IFN-β mouse ELISA kits according to the manufacturer's instructions.

### Luciferase assay

Cells were plated in 24-well plates and transfected with reporter gene plasmids (firefly luciferase) and pRL-TK (Renilla luciferase), together with different plasmids. Cells were treated with SeV, VSV or Poly (I: C) stimulation for the indicated time and collected. Luciferase activity was measured with Dual-Luciferase Assay according to the manufacturer's instructions with a Luminoskan Ascent luminometer (Thermo Fisher Scientific). The relative Reporter gene luciferase activity was determined by normalization of the firefly luciferase activity to Renilla luciferase activity. Data were shown as fold induction over empty vector-transfected controls.

### Viral infection *in vitro* and *in vivo*

Cells were infected with SeV, VSV, BEFV or H1N1 for the indicated times. For *in vivo* viral infection studies, age- and sex-matched Sec10^fl/fl^CRE-ER- and Sec10^fl/fl^CRE-ER+ mice were injected intraperitoneally with tamoxifen (50 μg/g body weight, dissolved in corn oil) for five consecutive days. After 7 days without treatment, mice were infected with SeV(1×10^8^PFU/mouse) via nasal instillation and monitored for body weight and survival status. Lungs from control or virus infected mice were dissected, fixed in 4% paraformaldehyde, embedded in paraffin, sectioned, stained with H&E solution, and examined by light microscopy for histological changes. IFN-β production in serum was measured by ELISA. The mRNA expression of *Ifnb*, *Isg15, Cxcl10, Ccl5* and SeV *NP* in the lung, liver and spleen was detected via real-time qPCR. The expression RIG-I and SeV NP protein in the lung, liver and spleen was determined by immunoblot assay.

### Virus challenge and TCID_50_ assay for detection of virus replication

Sec10 overexpression or knockdown/knockout cells were infected with SeV, BEFV and H1N1 for the indicated time. Subsequently, samples were collected and underwent three cycles of freeze-thawing and were subjected to TCID_50_ assay in 96-well plates. The virus titers, expressed as lgTCID_50_/mL, were calculated by the Reed-Muench method.

### Chromatin immunoprecipitation (ChIP)

The ChIP assay procedure was performed using SimpleChIP^TM^ Enzymatic Chromatin IP Kit as previously described^48^. Briefly, cells were cross-linked with 1% formaldehyde at room temperature for 10 min before reactions were quenched with 125 mM glycine at room temperature for 5 min. Nuclear extracts were sonicated with Covaris E220 for 660 s. After preclearing with normal IgG for 1 h, the sonicated cell lysates were incubated with anti-ATF4-linked beads and incubated for at least 6 hours for the IP assay. After successively washing with the indicated buffers, chromatin was eluted from the protein/DNA complex and digested with proteinase K and RNaseA at 65°C overnight to reverse cross-links. The freed DNA was purified with PCR Purification kit and subjected to PCR analysis. All sequences of primers for ChIP-PCR are listed in Supplemental Table 1.

### Protein inhibition assay

For protein degradation inhibition assays, HeLa cell lines with Sec10-expressing or vector control (2×10^5^ cells) were infected with SeV or VSV (MOI of 1). At 12 h postinfection, cells were treated with DMSO, MG132 (20 μM), Baf A1 (0.2 μM), CQ (50 μM) or Z-VAD (10 μM). Cells were lysed with RIPA buffer and subsequently analyzed by western blot assays.

### Quantification and statistical analysis

All graphs and statistical analysis were generated with GraphPad Prism8.0 software. All quantitative data obtained from repeated experiments are presented as the mean ± standard deviation (S.D). The statistical significance of the differences between the groups was calculated by two-tailed Student's t test. or one-way or two-way ANOVA as indicated, and the survival curves were analyzed using the log-rank (Mantel-Cox) test. Ns, not significant. Differences were considered statistically significant when values of P < 0.05. *P < 0.05; **P < 0.01; ***P < 0.001.

## Supplementary Material

Supplementary figures and tables.
