# Supplementary material for: Sec10 negatively regulates antiviral immunity by downregulating NRF2-ATF4-RIG-I axis
Source: Int J Biol Sci. 2025 Sep 3;21(13):5744–61. doi: 10.7150/ijbs.117430 (PMC12509678; doi:10.7150/ijbs.117430)
Supplement: Supplementary file 1 — Supplementary figures and tables. [file ijbsv21p5744s1.pdf]

Supplementary Materials for

Sec10 negatively regulates antiviral immunity response by downregulating NRF2-ATF4-RIG-I axis

Peili Hou et al.

***Corresponding author: Hongmei Wang: hongmeiwang@sdnu.edu.cn (H.W.); Hongbin He: hongbinhe@sdnu.edu.cn (H.H.)**

Table S1. Oligonucleotides used in this study

Primer name	Primers Sequences (5' to 3')	Application
Sec10-HA-F	CCGCTCGAGCGG GCCACCATGGCTACCACGGCCGAGCT	pLVX-Sec10-HA
Sec10-HA-R	CGGGATCCCGTCAAGCGTAATCTGGAACATCGTATGG GTAGCTGAAGTGTCGAGCAA	
ATF4-Flag-F	CCGGAATTCGCCACCATGACCGAAATGAGCTTCCTG	pCMV-Flag-ATF4
ATF4-Flag-R	GGGAAGAAAAGGGTCCCCGATTACAAGGACGACGAT GACAAGTAGGGATCCGCG	
NRF2-HA-F	CGCGGGCCCAGGCCCGAATTCATGATGGACTTGGAGC TGCCG	pCMV-HA-NRF2
NRF2-HA-R	ATGGGTAGCCGGTACCTCGAGGGTTTTTCTTAACATCT GGCTT	
RIG-I (-1113-1108)-F	CAAAGGTCCCAAGGCTATC	ChIP PCR for RIG-I promotor
RIG-I (-1113-1108)-R	TTGTTGTGGCGTGAGATG	
RIG-I (-1700-1695)-F	ACCAATGTGTCTATCTAC	ChIP PCR for RIG-I promotor
RIG-I (-1700-1695)-R	CAGGGTAAGTCTATGTTT	
RIG-I-M1-F	GCGTGCTAGCCCGGGCTCGAGCAGTGGTGCAATCTCG GCTC	$pGL_{3.0}$- RIG-I Promotor (Full-length)
RIG-I-M1-R	CAGTACCGGAATGCCAAGCTTCCCGCAGGCTGTGCCT CA	
RIG-I-M2-F	GCGTGCTAGCCCGGGCTCGAGCAGTGGTGCAATCTCG GCTC	$pGL_{3.0}$- RIG-I Promotor (-1000 to -1)
RIG-I-M2-R	CAGTACCGGAATGCCAAGCTTACTTCCTACTACTTTGA	

	CAATACCATTGT	
RIG-I-M3-F	GCGTGCTAGCCCGGGCTCGAGCAGTGGTGCAATCTCGGCTC	pGL$_{3.0}$- RIG-I Promotor (-500 to -1)
RIG-I-M3-R	CAGTACCGGAATGCCAAGCTTGCTGGAGTGCAGTGGCTATTCA	
RIG-I-M4-F	GCGTGCTAGCCCGGGCTCGAGCAGTGGTGCAATCTCGGCTC	pGL$_{3.0}$- RIG-I Promotor (-250 to -1)
RIG-I-M4-R	CAGTACCGGAATGCCAAGCTTGATTGGCTGGGTGTGGTGG	
RIG-I-M5-F	GCGTGCTAGCCCGGGCTCGAGCAAGAGTATCCTGTGGAGCACAGT	pGL$_{3.0}$- RIG-I Promotor (-2159 to -1000)
RIG-I-M5-R	CAGTACCGGAATGCCAAGCTTCCCGCAGGCTGTGCCTCA	
RIG-I-Mut1-F	ACGGTGC**CTCC**AATTAGAAATATGGAAAAAGTGAACCTG	pGL$_{3.0}$- RIG-I Promotor Mutants 1
RIG-I-Mut1-R	TCTAATT**GGAG**GCACCGTTTGTTGAAAACTTTTC	
RIG-I-Mut2-F	CCTGAAAAT**CT**CATC**CC**TATAAGTGTCTGCTCCTCAAAAGACAC	pGL$_{3.0}$- RIG-I Promotor Mutants 2
RIG-I-Mut2-R	A**GG**GATG**AG**ATTTTCAGGTTTTTCTTCTGTTTGTTG	
RIG-I-Mut3-F	TGGA**CT**ACTT**CC**TTCAGTCATTTTGGACAACAGGTG	pGL$_{3.0}$- RIG-I Promotor Mutants 3
RIG-I-Mut3-R	CTGAA**GG**AAGT**AG**TCCAGTTTACATTCCCACCCG	
shSMURF1	GCCCAGAGATACGAAAGAGAT	Knockdown of SMURF1 gene
siNC	UUCUCCGAACGUCACGUTT	Scrambled siRNA as negative control
siSec10:	GGUGAAAUCUCCAGAAUGA	Knockout of Sec10 gene
siATF4:	CCAAAUAGGAGCCUCCCAUTT	Knockout of ATF4 gene
siNRF2:	CCGGCATTTCACTAAACACAA	Knockout of NRF2 gene
siKeap1	GGGCGUGGCUGUCCUCAAUTT	Knockout of Keap1 gene
H-IFNB-F	GGACAGGATGAACTTTGACA	RT-qPCR for *IFNB* gene (Human)
H-IFNB-R	AGACATTAGCCAGGAGGTT	
H-ISG15-F	TCTGAGCATCCTGGTGAG	RT-qPCR for

H-ISG15-R	GAAGGTCAGCCAGAACAG	*ISG15* gene (Human)
H-CXCL10-F	ACTGCCATTCTGATTTGCTGCC	RT-qPCR for *CXCL10* gene (Human)
H-CXCL10-R	GCTGATGCAGGTACAGCGTACA	
H-CCL5-F	ATGAAGGTCTCCGCGGCACGCCT	RT-qPCR for *CCL5* gene (Human)
H-CCL5-F	CTAGCTCATCTCCAAAGAGTTG	
H-β-actin	CGAGAAGATGACCCAGAT	RT-qPCR for housekeeping gene (Human)
H-β-actin	GATAGCACAGCCTGGATA	
RIG-I-F	GATGAGATTGAGCAAGAT	RT-qPCR for *RIG-I* gene (Human)
RIG-I-R	TTGTAAGATGAAGCAGAG	
SeV NP-F	TCAGGAGGAGGTGCTGTTATC	RT-qPCR for *NP* gene (SeV)
SeV NP-R	TTGGGCCTAGTACGAACACTG	
H1N1 NP-F	TGTATGGACCTGCCGTAG	RT-qPCR for *NP* gene (H1N1)
H1N1 NP-R	TGCTTCTCAGTTCAAGTGTA	
BEFV N-F	GTCACCTTATTCATCAATCTCT	RT-qPCR for *N* gene (BEFV)
BEFV N-R	TGCTCACTAACCATACGA	
M-β-actin -F	CCACACCCGCCACCAGTTCG	RT-qPCR for housekeeping gene (Human)
M-β-actin -R	TACAGCCCGGGGAGCATCGT	
M-Ifnb-F	CAGCTCCAAGAAAGGACGAAC	RT-qPCR for *Ifnb* gene (Mouse)
M-Ifnb-R	GGCAGTGTAACTCTTCTGCAT	
M-Isg15-F	AGAAGCAGATTGCCCAGAAG	RT-qPCR for *Isg15* gene (Mouse)
M-Isg15-R	TGCGTCAGAAAGACCTCATAGA	
M-Cxcl10 -F	AGTAATGGATCTGTTAATC	RT-qPCR for *Cxcl10* gene (Mouse)
M-Cxcl10-R	AATTCTTGATGGTCTTAG	
M-Ccl5-F	TTGTCTTTATCACCAGGAA	RT-qPCR for *Ccl5* gene (Mouse)
M-Ccl5-R	AAGCGTTGATGTACTCTC	
B-IFNB-F	CCTGTGCCTGATTTCATCATGA	RT-qPCR for *IFNB* gene (Bovine)
B-IFNB-R	GCAAGCTGTAGCTCCTGGAAAG	

B-ISG15-F	GGAGGCCCATGGATGATG	RT-qPCR for *ISG15* gene (Bovine)
B-ISG15-R	CCGAAGACGTAGATTCATGAACAC	
B-CXCL10-F	TTCTGCCTTATCCTTCTGA	RT-qPCR for *CXCL10* gene (Bovine)
B-CXCL10-R	TTATGCCTCTTTCCGTGTT	
B-CCL5-F	GCCCTGCTGCTTTGCCTAT	RT-qPCR for *CCL5* gene (Bovine)
B-CCL5-F	GCACTTGCTGCTGGTGTAG	
B-β-actin	GATGAGATTGGCATGGCTTTA	RT-qPCR for housekeeping gene (Bovine)
B-β-actin	AACCGACTGCTGTCACCTTC	
Loxp-F1	CAGTCTTTGGGTTGTGTTTTAAGC	Genotyping for $Sec10^{fl/fl}$ mice
Loxp-R1	CCTGTTTCACTATCCAGGTTACGG	
Loxp-R2	GCCTAATGTACGCTCTAAAGTCTTC	
Cre-F	CATATTGGCAGAACGAAAACGC	Genotyping for CRE-ER mice
Cre-R	CCTGTTTCACTATCCAGGTTACGG	

Note: Bold, mutation sites. H: Human; M: Mouse; B: Bovine.

Table S2. KEY SOURCES TABLE

**Reagent or resource**	**Source**	**Identifier**
**Antibodies**		***Cat#***
Mouse anti- Sec10	Santa Cruz	sc-514802
Rabbit anti-Phospho-TBK1 (Ser172)	CST	5483
Rabbit anti-HA	CST	2999
Rabbit anti-DYKDDDDK/Flag	CST	14793
Normal Rabbit IgG (ChIP Formulated)	CST	2729
Rabbit anti-RIG-I	Abways	CY6992
Rabbit anti-ISG15	Abways	CY9357
Rabbit anti-IFITM3	Abways	CY7091
Rabbit anti-ATF4	Abways	CY5873
Rabbit anti- RXRA	Abways	CY8640
Rabbit anti-N2RF1	Abways	CY7048
Rabbit anti-STAT5A	Abways	CY5056
Rabbit anti-Phospho-IRF3	Abways	CY6575
Rabbit anti-β-actin	Abclonal	AC038
Rabbit anti-H1N1 M1	SinoBiological	40010-T62
Rabbit anti- NRF2	Wanleibio	WL02135

Mouse anti-DYKDDDDK/Flag	MBL	M183-3L
Mouse anti-BEFV N	In this study	N/A
Mouse anti-SeV NP	In this study	N/A
AffiniPure Goat Anti-Mouse IgG (H+L)	Jackson Immunoresearch	AB2338447
AffiniPure Goat Anti-Rabbit IgG (H+L)	Jackson Immunoresearch	AB2337913
**Bacterial and Virus Strains**		
DH5α Competent *E. coli*	NEB	N/A
BEFV	In this study	N/A
SeV	In this study	N/A
VSV	Changchun Veterinary Research Institute	N/A
Influenza A/Changchun/01/2009 (H1N1)	Changchun Veterinary Research Institute	A/Changchun/01/2009
**Chemicals and Recombinant Proteins**		
Dimethyl sulfoxide (DMSO)	Med Chem Express	HY-Y0320
Chloroquine diphosphate salt (CQ)	Med Chem Express	HY-17589A
Z-VAD-FMK	Med Chem Express	HY-16658B
MG132	Med Chem Express	HY-13259C

Continued

**Reagentor resource**	**Source**	**Identifier**
5'ppp-dsRNA	InvivoGen	tlrl-3prna
Mouse M-CSF Recombinant Protein	Novoprotein,	CJ46
LMW Poly(I:C)	InvivoGen	tlrl-picwlv
Tamoxifen	Med Chem Express	HY-13757A
4-hydroxytamoxifen	Sigma	H6278
Attractene Transfection Reagent	QIAGEN	301007
T4 polynucleotide kinase	NEB	M0201S
DMEM	Viva Cell	C3113-0500
Puromycin	Solarbio	P8230
FBS	ExCell Bio	FSP500
Penicillin and streptomycin	Procell	PB180120
RIPA Buffer	New Cell and Molecular	WB3100
Halt™ Protease Inhibitor Cocktail, EDTA-Free (100×, P001)	New Cell & Molecular	P002
**Critical Commercial Assays**		
SuperScriptIII first-strand synthesis kit	Accurate	AG11706
Duo-Lite TM Luciferase Assay System	Vazyme Biotech Co. Ltd	DD1205
Total RNA isolation kit	FOREGENE	RE-03111
Mouse IFN-β ELISA Kit	Lianke	EK2236
QuickChange Lightning Site-directed	Agilent	210518

Mutagenesis kit		
Master Mix PCR	Vazyme	P525-01
SDS-PAGE gels	Epizyme	PG113
PCR Purification kit	Tiangen	DP209
SYBR™ Green PCR Master Mix	Accurate	AG11739
Chemiluminescence Detection Kit	Shandong Sparkjade Biotechnology	ED0015-C
Chromatin Immunoprecipitation (ChIP) Kit	Beyotime	P2078
**Experimental Models: Cell lines**		
HeLa cells	ATCC	CCL-2
HEK293T cells	ATCC	CRL-1537
A549 cells	ATCC	CCL-185
MDBK cells	ATCC	CCL-22
IFNAR1-KO cell lines	In this study	N/A
STUB1-KO cell lines	In this study	N/A
shSMURF1 cell lines	In this study	N/A
Bone Marrow Derived Macrophages	In this study	N/A
Peritoneal Macrophages	In this study	N/A
**Oligonucleotides**		
Please see Supplementary Tables 1 for details		
**Software and Algorithms**		
GraphPad Prism Software	GraphPad Prism Software, Inc.	N/A
Primer Premier 6	GraphPad Software, Inc	N/A
MEGA6	Gene STAR	N/A
CRISPR direct	http://crispr.dbcls.jp/	N/A
JASPAR CORE database program	http://jaspar.genereg.net/	N/A
PROMO database	https://alggen.lsi.upc.es/	N/A
Continued		
**Reagentor resource**	**Source**	**Identifier**
GeneCards	https://previous.genecards.org/	N/A

Supplementary Materials for

Sec10 negatively regulates antiviral immunity response by downregulating NRF2-ATF4-RIG-I axis

Peili Hou et al.

***Corresponding author: Hongmei Wang: hongmeiwang@sdnu.edu.cn (H.W.); Hongbin He: hongbinhe@sdnu.edu.cn (H.H.)**

Supplemental Figure

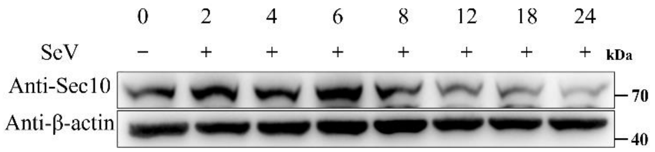

Fig. S1. SeV infection regulates the expression of Sec10.

HeLa cells were infected with SeV for the indicated time points, and then the expression of Sec10 was detected by immunoblotting analysis.

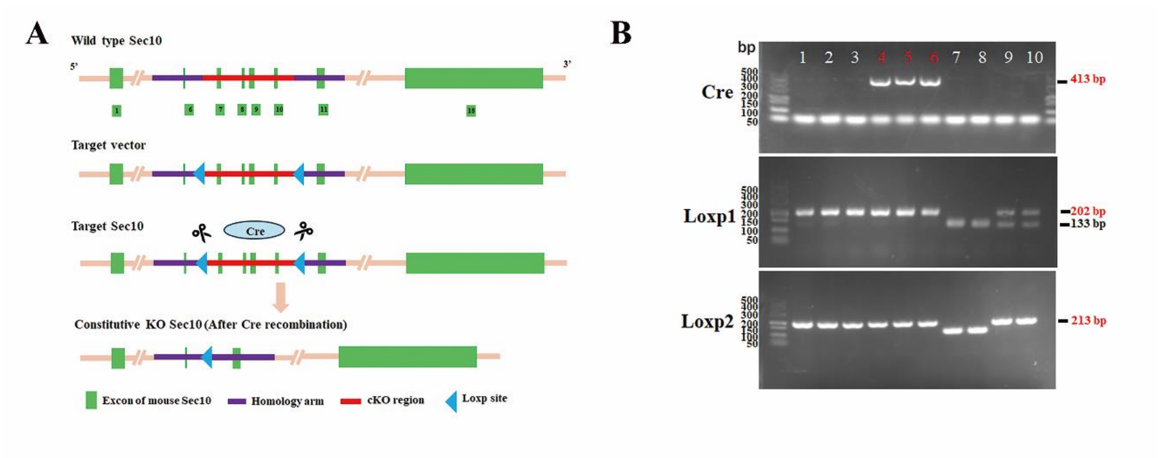

Fig. S2. Schematic diagram of the construction and identification of $Sec10^{fl/fl}$CRE-ER+ mice.

(A)Scheme showing the CRISPR/Cas9-mediated genome editing of the Sec10 gene locus. (B) Identification of $Sec10^{fl/fl}$CRE-ER+ mice. PCR analysis of the genotypes of $Sec10^{fl/fl}$CRE-ER+ (Lane 1-4) and $Sec10^{fl/fl}$CRE-ER- (Lane 5-7) mice (PCR 1: Cre positive, 413 bp. PCR 2: loxp 1, $Sec10^{fl/fl}$ positive, 202 bp. PCR 3: loxp 2, $Sec10^{fl/fl}$CRE-ER positive, 213 bp).

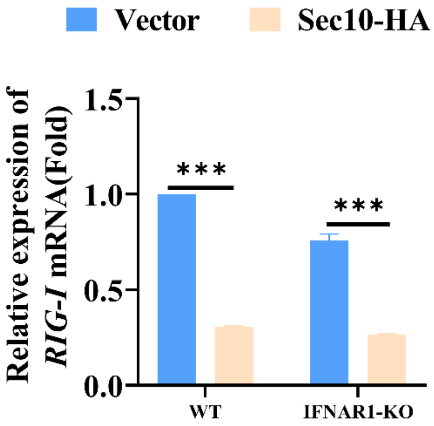

Fig. S3. Real time PCR analysis the *RIG-I* mRNA expression upon Sec10 expression with IFN-AR1 deficiency.

Sec10-HA or vector control was overexpressed in wild-type and IFNAR1-KO cell lines, followed by SeV infection for 12 hours. Then the *RIG-I* mRNA expression was conducted by Real time PCR analysis. Data are presented as mean±S.D from three independent experiments, two-way ANOVA for date analysis; ***P<0.001.

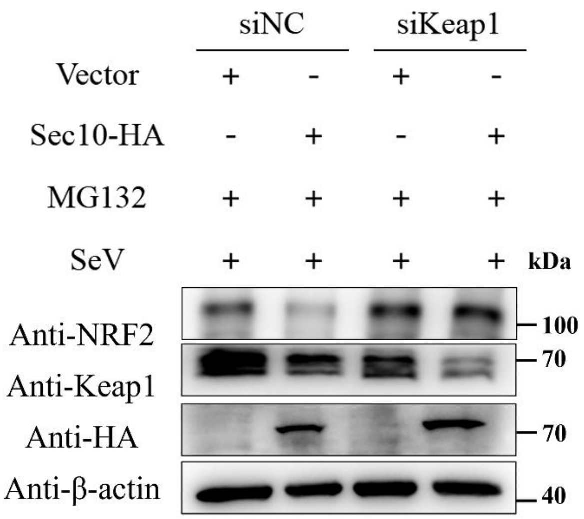

Fig. S4. The effect of Keap1 knockdown on Sec10-mediated regulation of NRF2 expression. Sec10-HA or vector control overexpressing HeLa cells were transfected with siNC or siKeap1 in HeLa cells, followed by SeV infection for 12 hours. Then the indicated protein expression was conducted by immunoblotting analysis.

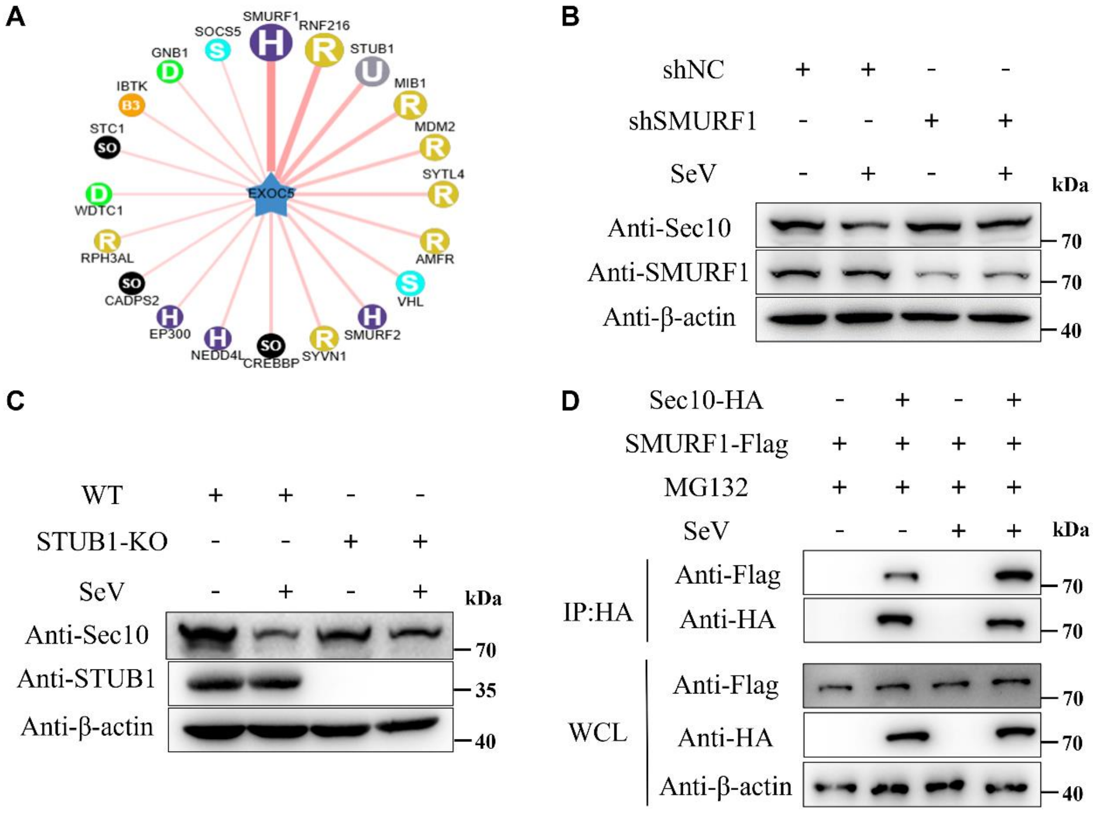

Fig. S5. SeV infection downregulates Sec10 via the SMURF1-mediated ubiquitin-proteasome pathway.

(A) The potential E3 ubiquitin ligases were predicted by the UbiBrowser software (http://ubibrowser.ncpsb.org). (B) HeLa cells were transfected with shNC or shSMURF1, followed by Mock or SeV infection for 12 h, and then Sec10 expression was determined by immunoblotting analysis. (C) WT and STUB1-KO cell lines were infected or uninfected with SeV, and then Sec10 expression was determined by immunoblotting analysis. (D) SMURF1-Flag were transfected with vector or Sec10 -HA in the presence of MG132 treatment, respectively, followed by SeV infection or mock infection for 12 h. Co-immunoprecipitation (co-IP; with anti-HA) and immunoblotting analysis of the indicated protein expression.

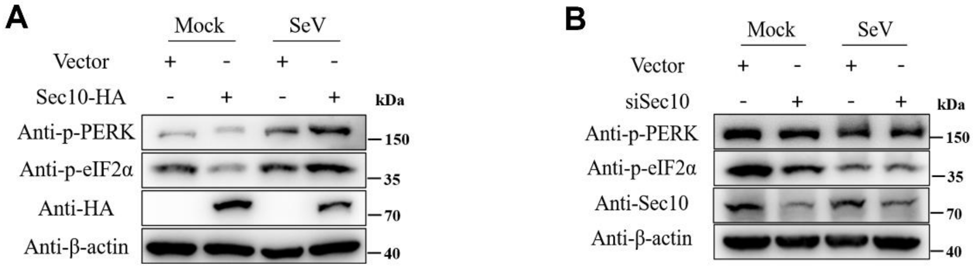

Fig. S6. Effect of Sec10 on the ER stress-induced PERK-eIF2α signaling pathway.

HeLa cells were transfected with either Vector control, Sec10-HA plasmids, or siNC, siSec10 followed by SeV infection for the indicated time points. The indicated protein expression levels were analyzed by immunoblotting.
